# Estradiol- and Progesterone-Associated Changes in microRNA-Induced Silencing and Reduced Antiseizure Efficacy of an Antagomir in Female Mice

**DOI:** 10.1523/ENEURO.0047-22.2023

**Published:** 2023-07-21

**Authors:** Durgesh Tiwari, Valerine Rajathi, Jeffrey K. Rymer, Lindsay N. Beasley, Amanda M. McGann, Alexander T. Bunk, Emma V. Parkins, McKenzie F. Rice, Katie E. Smith, David M. Ritter, Angela R. White, Carolyn M. Doerning, Christina Gross

**Affiliations:** 1 Division of Neurology, Cincinnati Children’s Hospital Medical Center, Cincinnati, Ohio 45229; 2 Department of Pediatrics, University of Cincinnati College of Medicine, Cincinnati, Ohio 45229; 3 Veterinary Services, Cincinnati Children’s Hospital Medical Center, Cincinnati, Ohio 45229

**Keywords:** epilepsy, female, microRNA, miR-324-5p, Kv4.2

## Abstract

About one-third of individuals living with epilepsy have treatment-resistant seizures. Alternative therapeutic strategies are thus urgently needed. One potential novel treatment target is miRNA-induced silencing, which is differentially regulated in epilepsy. Inhibitors (antagomirs) of specific microRNAs (miRNAs) have shown therapeutic promise in preclinical epilepsy studies; however, these studies were mainly conducted in male rodent models, and research into miRNA regulation in females and by female hormones in epilepsy is scarce. This is problematic because female sex and the menstrual cycle can affect the disease course of epilepsy and may, therefore, also alter the efficacy of potential miRNA-targeted treatments. Here, we used the proconvulsant miRNA miR-324-5p and its target, the potassium channel Kv4.2, as an example to test how miRNA-induced silencing and the efficacy of antagomirs in epilepsy are altered in female mice. We showed that Kv4.2 protein is reduced after seizures in female mice similar to male mice; however, in contrast to male mice, miRNA-induced silencing of Kv4.2 is unchanged, and miR-324-5p activity, as measured by the association with the RNA-induced silencing complex, is reduced in females after seizure. Moreover, an miR-324-5p antagomir does not consistently reduce seizure frequency or increase Kv4.2 in female mice. As a possible underlying mechanism, we found that miR-324-5p activity and the silencing of Kv4.2 in the brain were differentially correlated with plasma levels of 17β-estradiol and progesterone. Our results suggest that hormonal fluctuations in sexually mature female mice influence miRNA-induced silencing and could alter the efficacy of potential future miRNA-based treatments for epilepsy in females.

## Significance Statement

MicroRNA (miRNA)-induced silencing is currently investigated in preclinical studies as a potential novel class of treatment target for epilepsy; however, surprisingly little is known about whether and how miRNA-induced silencing is regulated by biological sex and whether miRNA therapeutics are as effective in females as they are in males. This study shows that miRNA silencing of the potassium channel Kv4.2 and the functional activity of its targeting miRNA, miR-324-5p, change with plasma levels of estrogens and progesterone and that an inhibitor of miR-324-5p is less effective in female mice to suppress seizures. These findings are significant as they suggest miRNA-induced silencing as a novel molecular mechanism contributing to sex differences in epilepsy that could impact future therapy development.

## Introduction

Approximately 30% of women and men living with epilepsy experience seizures despite treatment and despite the rapid rise in approved drug treatments over the last 3 decades (Chen et al., 2018). Conceptually novel treatment strategies, including new drug targets, are urgently needed. One example of a promising new treatment target is microRNA (miRNA)-induced silencing. MicroRNAs are small noncoding RNAs that can regulate entire biological pathways through controlling the translation or stability of many different mRNAs (Bartel, 2018). MicroRNA expression is differentially regulated in epilepsy and inhibiting select microRNAs with short antisense sequences (antagomirs) reduces seizure susceptibility and frequency in epilepsy mouse and rat models (Brennan and Henshall, 2020).

MicroRNAs recruit their mRNA targets to the RNA-induced silencing complex (RISC) where the mRNAs are functionally silenced. Previous work has shown that RISC association of miR-324-5p and its mRNA target coding for the voltage-gated potassium channel Kv4.2 is increased after seizure and in epilepsy (Gross et al., 2016; Tiwari et al., 2019), suggesting elevated activity of miR-324-5p and increased silencing of Kv4.2. Inhibition of miR-324-5p with antagomirs increases expression of functional Kv4.2 channels and reduces susceptibility to kainic acid (KAI)-induced seizures and seizure frequency in male mouse models, which is partially mediated through Kv4.2 (Gross et al., 2016; Tiwari et al., 2019). Antagomirs are in clinical trials for cancer and other diseases (Winkle et al., 2021), and there is thus reasonable hope that antagomir-based therapies could also soon be tested in human epilepsy trials.

As is often the case in preclinical studies, however, data on the efficacy of antagomir treatment in female rodent models of epilepsy are scarce. The vast majority of preclinical antagomir studies in mouse or rat models of epilepsy have used male mice and did not assess their effects on female rodents. This could be a major problem for translating antagomir therapies into clinical studies because miRNA expression can differ between male and female brains (Murphy et al., 2014) and it is well known that biological sex affects the disease course of epilepsy (Christian et al., 2020). For example, seizure susceptibility can fluctuate during the menstrual cycle (also known as catamenial epilepsy; Frank and Tyson, 2020), and female sex hormones, such as estrogens and progesterone, alter neuronal excitability. In general, estrogens are believed to promote excitation, and progesterone to reduce excitation (Woolley, 1998; Reddy et al., 2010); however, the relationship between these hormones and neuronal excitability is most likely more complex than previously thought (Kapur and Joshi, 2021). Moreover, clinical trials with progesterone in women with epilepsy have not shown the expected success (Herzog et al., 2012). This demonstrates the need for further analyses of the molecular mechanisms driving neuronal excitability in male and female organisms to elucidate how epilepsy treatment efficacy differs depending on sex.

So far, it is not known how female sex and estrous cycle affect miRNA-induced silencing in epilepsy and whether the effectiveness of antagomir treatments in epilepsy is altered depending on biological sex. To address this gap, here, we analyzed miR-324-5p and its target, the voltage-gated potassium channel Kv4.2, in a female seizure mouse model. We show that miR-324-5p antagomirs do not consistently delay seizure onset in female mice, as previously observed in male mice (Gross et al., 2016). Moreover, the antagomirs do not consistently increase Kv4.2 expression in female mice. As a potential mechanism, we identify correlations between plasma progesterone and/or 17β-estradiol levels and miRNA-induced silencing of Kv4.2 in the hippocampus and cortex. Our results suggest that miRNA-dependent regulation of neuronal function is associated with the levels of female sex hormones, which could contribute to female-specific etiologies of epilepsy and may have an impact on the efficacy of future antagomir-based treatments in epilepsy.

## Materials and Methods

### Mice

**Figure 1.**
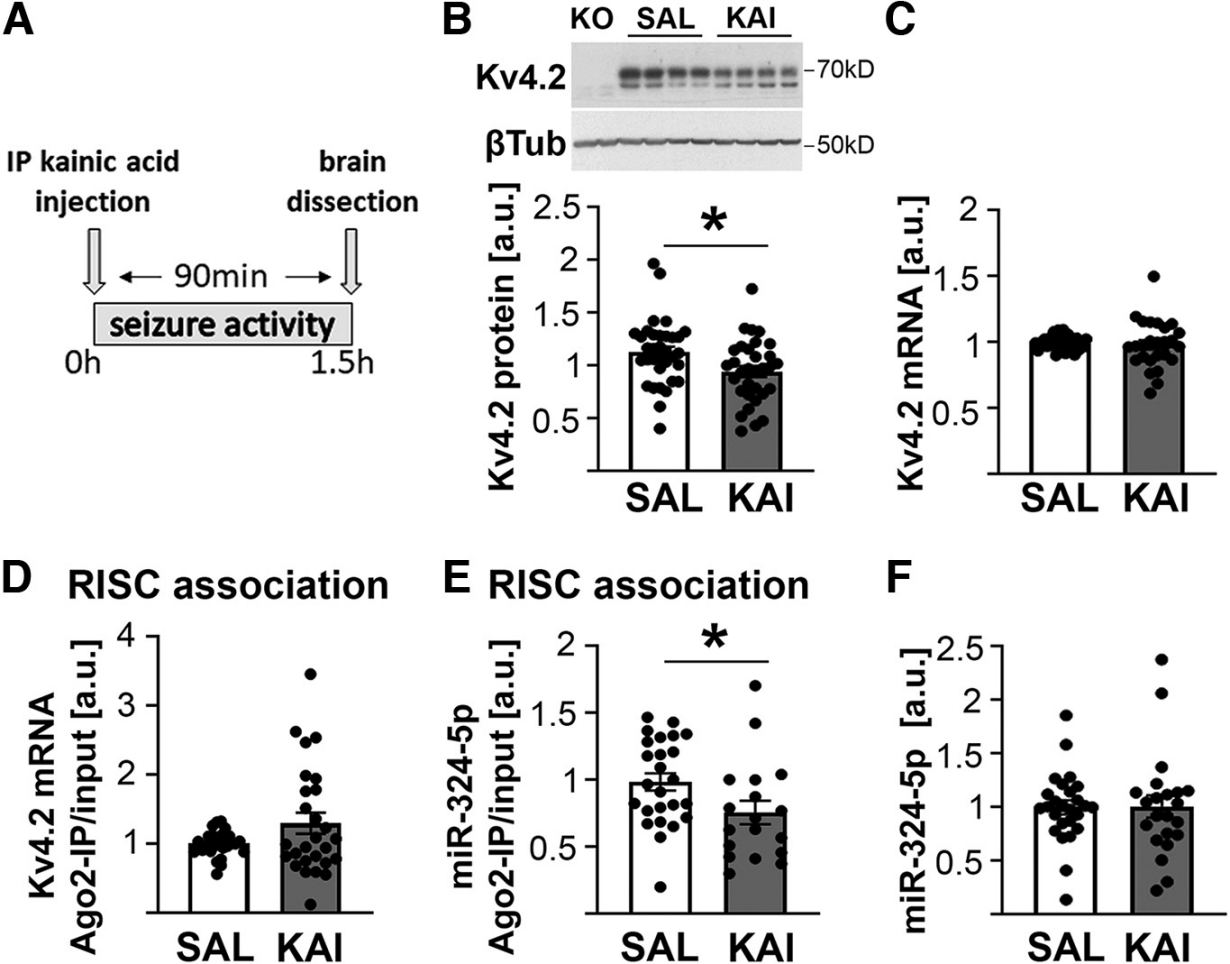
Kv4.2 protein levels in the hippocampus are reduced 90 min following kainic acid-induced seizure, but no changes in miRNA-mediated silencing of Kv4.2 are detected. ***A***, Timeline of kainic acid injection and tissue harvest. ***B***, ***C***, Hippocampal Kv4.2 protein levels are significantly reduced in female mice 90 min following injection of 15 mg/kg, i.p., kainic acid (***B***; unpaired *t* test, *p = 0.020; *n*(SAL) = 34, *n*(KAI) = 32), whereas mRNA levels are not significantly different (***C***; Mann–Whitney test, *p* = 0.641; *n*(SAL) = 27, *n*(KAI) = 26; 2 statistical outliers were removed from the saline group, and 3 statistical outliers were removed from the kainic acid group), similar to those observed in male mice (Gross et al., 2016). Kv4.2 protein levels were normalized to β3-tubulin levels on the same blot, and Kv4.2 mRNA was normalized to Gapdh mRNA in the same samples. ***D***, In contrast to male mice, RISC association of Kv4.2 mRNA is not significantly increased after kainic acid in the hippocampus of female mice (Mann–Whitney test, *p* = 0.741; *n* = 27/group). ***E***, ***F***, Hippocampal RISC association of the Kv4.2-targeting miRNA miR-324-5p is reduced in female mice following kainic acid administration (***E***; Mann–Whitney test, *p = 0.038; *n*(SAL) = 25, *n*(KAI) = 18; 2 statistical outliers removed from the kainic acid group), while total miR-324-5p levels are unchanged (***F***; Mann–Whitney test, *p* = 0.735; *n*(SAL) = 26, *n*(KAI) = 22; 2 statistical outliers removed from the kainic acid group). In ***F***, miR-324-5p total levels were normalized to RU19 or miR-191 in the same samples. SAL, saline; KAI, kainic acid; a.u., arbitrary units. Bars and error bars represent the mean ± SEM. Analyses in cortical tissue are shown in Extended Data Figure 1-1.

Female C57BL/6J wild-type (WT) mice between 6 and 8 weeks of age were used for this study. Mice were generated by breeding using C57BL/6J WT breeding pairs that were originally obtained from The Jackson Laboratory (stock #000664; RRID:IMSR_JAX:000664). Experimental mice were weaned from the dam at postnatal day 21 (P21) to P28 and were group housed with up to three female littermates (maximum, four per cage) in a standard cage on a 14 h light/10 h dark cycle with food and water provided *ad libitum.* Mice were single housed after surgeries to avoid chewing off sutures or electrodes. All experiments were performed during the light cycle. A total of 158 female WT mice were used for this study. Thirty-nine female mice from 10 different litters (19 injected with saline, 20 injected with kainic acid) were used for vaginal cytology, blood collection, and brain dissection on 6 separate experimental days followed by biochemical assays. A second cohort of 31 female mice from three different litters (16 injected with saline, 15 injected with kainic acid) were used for blood collection and brain dissection on two separate experimental days followed by biochemical assays. A total of 28 female mice from 10 different litters were used for intracerebroventricular stereotaxic injections of miR-324-5p-specific or scrambled (scr) antagomir and intraperitoneal injections of saline or kainic acid (seven per condition) followed by brain dissection and biochemical assays. A total of 24 mice from five different litters were implanted with cortical electrodes and intracerebroventricularly injected with miR-324-5p-specific or scrambled antagomir (12 per condition) followed by intraperitoneal injection with kainic acid the next day for EEG seizure analysis and brain dissection. Twenty female mice from more than two litters were used for ovariectomies and saline or kainic acid treatment 2 weeks later (10 mice each) and then were used for tissue collection and biochemical analyses. Sixteen mice were used for artificial CSF (ACSF) intracerebroventricular injections (sham control) followed by intraperitoneal injection of kainic acid and brain tissue collection 24 h later. Mice that were not included for analyses because of experimental failure or because they were identified as a statistical outlier using the Rout method (see subsection Experimental design and statistical analyses) are listed under the respective method sections or in the figure legends. Male littermates were used for other studies. Note that all mice used for correlation analyses were also used in pairwise comparisons to avoid bias (Fig. 1, Extended Data Fig. 1-1), and a subset was used for analyses seen in Figure 7 and Extended Data Figure 7-1. The sample sizes in the pairwise comparisons are higher than those shown in the correlation analyses because a few progesterone and several estradiol ELISAs failed and could not be repeated because of the insufficient amounts of blood recovered. All animal procedures were approved by the Institutional Animal Care and Use Committee of Cincinnati Children’s Hospital Medical Center and complied with the *Guidelines for the Care and Use of Laboratory Animals*.

10.1523/ENEURO.0047-22.2023.f1-1 Figure 1-1 Kv4.2 protein levels and microRNA-induced silencing of Kv4.2 mRNA are unchanged in the cortex of female mice 90 min following kainic acid-induced seizure. ***A***, Timeline of kainic acid injection and tissue harvest. ***B***, ***C***, Cortical Kv4.2 protein levels are unchanged in female mice 90 min following injection of 15 mg/kg, i.p., kainic acid (***B***; unpaired *t* test, *p* = 0.937, *n* = 32/group). Similarly, Kv4.2 mRNA levels are unchanged (***C***; unpaired *t* test, *p* = 0.372, *n* = 26; 3 statistical outliers removed from the saline group). Kv4.2 protein levels were normalized to β3-tubulin levels on the same blot, and Kv4.2 mRNA was normalized to Gapdh mRNA in the same samples. ***D***, RISC association of Kv4.2 mRNA is not changed after kainic acid in the cortex of female mice (unpaired *t* test, *p* = 0.226; *n*(SAL) = 29, *n*(KAI) = 30; 1 statistical outlier removed from kainic acid group). ***E***, ***F***, Cortical RISC association of the Kv4.2-targeting microRNA miR-324-5p shows a trend towards being reduced in female mice following kainic acid (***E***; unpaired *t* test, *p* = 0.062; *n*(SAL) = 32, *n*(KAI) = 29), and miR-324-5p levels are unchanged (***F***; unpaired *t* test, p = 0.838, *n*(SAL) = 31, *n*(KAI) = 27; 2 statistical outliers removed from the kainic acid group). In ***F***, miR-324-5p total levels were normalized to RU19 or miR-91 in the same samples. a.u., Arbitrary units. Bars and error bars represent the mean ± SEM. Analyses in hippocampal tissue are shown in Figure 1. Download Figure 1-1, TIF file.

### Reagents

The following antibodies were used in this study: mouse monoclonal anti-Ago2/eIFC2 (catalog #H00027161-M01, Abnova; RRID:AB_565459); rabbit polyclonal anti-Kv4.2 (catalog #21298–1-AP, Proteintech Group; RRID:AB_10733102); and rabbit polyclonal anti-β3-tubulin (catalog #USA-802001, BioLegend; RRID:AB_2564645). Secondary antibodies for Western blot detection with chemiluminescence were obtained from GE Healthcare Life Sciences. The following quantitative real-time PCR (qRT-PCR) primers were used: Kv4.2: forward, GCTTTGAGACACAGCACCAC; reverse, TGTTCATCGACAAACTCATGG; Kcnq2: forward, AAATCTGGACTCACCTTCAGGA; reverse, CACGGTCTGCCTTTACTTGG; Kcnq3: forward, CCAGCTGCGGAACTCATC; reverse, CAACCTGTTGGGGTTGGTAG; Gapdh: forward, CAAGGTCATCCATGACAACTTTG; reverse, GGGCCATCCACAGTCTTCTG; miR-324-5p: CGCATCCCCTAGGGCATTGGTGT; and RU19: GAGATCGTGTTACACTGTTGG. *In vivo* antagomirs were custom-made by Qiagen using the following sequences: miR-324-5p, ACCAATGCCCTAGG; and scrambled ACGTCTATACGCCCA. Both antagomirs had the same amount of locked nucleic acid (LNA)-modified nucleotides within the sequence as well as a partial phosphorothioate backbone. Kainic acid (catalog #0222, Tocris Bioscience) was dissolved at 2 mg/ml in sterile saline.

### Ovariectomies

Virgin female C57BL/6J mice were ordered from The Jackson Laboratory and allowed to acclimate to the facility for at least 3 d. Mice were 6 weeks of age at the time of ovariectomy. Briefly, mice were anesthetized with isoflurane, and the fur was removed on the lower dorsal back. Eye lubricant was applied, and pre-emptive local anesthetics were given (bupivacaine, 0.25%; 2 mg/kg; Hospira). The mouse was positioned prone, and the skin was aseptically prepared. Using the iliac crest as a landmark, a dorsal midline skin incision was made. The first ovary was located, a small underlining incision was made through the abdominal wall, and the ovary was exteriorized using forceps. The ovary was then excised from the oviduct with uterine horn and fat pad using a crush-and-tear technique. After returning the severed oviduct, the uterine horn and fat pad were returned to the abdominal cavity. The body wall was closed using absorbable sutures. The procedure was then repeated on the other ovary, and the skin incision was closed with intradermal absorbable sutures. Postoperative analgesics (carprofen, 5 mg/kg; 1 mg/ml, once daily for a minimum of 48 h; Zoetis) as well as subcutaneous fluids (0.9% saline; Hospira) were provided as needed. Mice were allowed to recover for 2 weeks and were then injected with kainic acid, as described below. One mouse required repair of incisional dehiscence following ovariectomy and was excluded from the analysis.

### Intracerebroventricular antagomir injection and cortical EEG electrode implantation

Six- to 8-week-old female C57BL/6J mice were bilaterally injected into brain ventricles with miR-324-5p antagomir or scrambled control (0.5 nmol in 2 μl sterile ACSF/side). All surgeries were performed under a deep plane of anesthesia induced and maintained with isoflurane, and pain medication was administered presurgery and postsurgery. For intracerebroventricular antagomir injections, two burr holes were drilled anteroposterior (AP) –0.3 mm, lateral (L) ±1.0 mm, and ventral (V) 2.0 mm from bregma, and antagomir was slowly injected over 5 min using a 5 μl Hamilton syringe. The needle was then left in place for 5 min and slowly retracted over 5 min. Some mice received cortical electrode implants during the same surgery. To this end, two additional burr holes were drilled into the skull at AP −2.5 mm and L ±2.0 mm from bregma without injuring the dura (Tse et al., 2014). A wireless transmitter (model TA11ETAF-10) from Data Sciences International was placed subcutaneously at the back of the mice, and the leads were placed into each burr hole on the dura mater (without penetrating it) for cortical surface EEG recordings. Dental cement (Lang Dental) was used to hold the setup in place. The skin incision was closed with surgical sutures (Covidien, Medtronic) and GLUture (Zoetis). An antibiotic ointment (RARO) was applied on top of the sutured incision. To facilitate recovery, mice were intraperitoneally injected with 1 ml of sterile saline, and kept on a heating pad and monitored until fully ambulatory again.

### Kainic acid injections and EEG recording

To induce seizures, mice were injected intraperitoneally with kainic acid (15 mg/kg body weight, 2 mg/ml in sterile saline solution). Some mice received an equal volume of intraperitoneal saline injections and served as seizure controls. Injections occurred between 1:00 and 4:00 P.M. in the afternoon to avoid effects of the circadian rhythm. Lights were turned off at 7:00 or 8:00 P.M., respectively (depending on daylight saving time). Therefore, experiments took place between 3 and 7 h before the start of the dark cycle. Mice were either naive at the time of injection, were ovariectomized, or had received stereotaxic intracerebroventricular injections of antagomirs with or without EEG implanting 24 h before kainic acid treatment. For those mice implanted with EEG electrodes, cages were placed on wireless receiver plates (model RPC1, Data Sciences International), and baseline EEG was recorded using DATAQUEST Advanced Research Technology system for ∼15 min before the kainic acid treatment started. Video/EEG recording was continued throughout the 90 min following kainic acid injections. EEG data were sampled at 500 Hz, providing readouts for frequencies between 1 and 200 Hz (maximal sampling rate of the wireless transmitter model TA11ETA-F10). At the same time, video was continuously recorded (Axis 221 Network Camera, Axis Communications) and synchronized with the EEG signal (Ponemah Video Software, Noldus). Ninety minutes after kainic acid (or saline) injection, all mice were killed, and cortices and hippocampi were dissected, flash frozen on dry ice, and stored at −80°C until further use. In addition, blood was collected by cardiac puncture from the non-EEG mice, transferred into 10 μl 0.5 m EDTA-containing Eppendorf tubes, centrifuged for 10 min at 1000 × *g* at 4°C, and the resulting supernatant (plasma) was stored at −80°C until further analysis.

### EEG analysis and behavioral seizure classification

The EEG recording data were analyzed using NeuroScore software (Data Sciences International). Seizure onset was defined as a sudden increase in EEG amplitude (>2× background) and frequency. In addition, onset of behavioral seizures was determined by observing the video recordings in parallel to EEG signal. Behavioral seizures were classified using a modified Racine scale. Class III behavioral seizures were characterized by head bobbling, vibrissae twitching, and tail raising, and class V seizure were characterized by loss of posture, rearing, and falling, and by whole-body clonus, “corkscrew” turning, and flipping. Class IV seizures were not included in the analysis because of their overlapping behavioral characteristics with class III and V seizures (Racine, 1972). Status epilepticus was defined as continuous EEG seizure activity that did not return to baseline for the remainder of the recording period. Of the 24 mice injected with antagomirs and implanted with electrodes, five mice were excluded from the entire experiment for the following reasons: two mice were excluded because of excessive subcranial bleeding, one mouse was excluded because it did not thrive following intracerebroventricular surgery, and two mice were excluded because they did not show behavioral or EEG seizures. In addition, one died shortly after kainic acid treatment and was thus only used for seizure latency analysis. One mouse lacked a detectable EEG signal because of transmitter failure and thus was not used for EEG-based analyses, and one had an EKG signal overlying the EEG signal and could thus not reliably be used to quantify seizure numbers and duration.

### Vaginal cytology to determine estrous stage

Naive female mice at 6–8 weeks of age that were used for kainic acid or saline injections received daily vaginal swabs for 5 d until the day of experiment. At the day of experiment, mice received a vaginal swab before kainic acid or saline treatment and at the time of killing. Vaginal swabs were performed using a cotton tip applicator wetted in phosphate buffer. Collected cells were transferred on microscope slides, dried, and immediately stained using Jorvet Dip Quick Stain (catalog #J-322, Jorgensen Laboratories). Estrous stages were defined as follows: proestrus, nucleated and cornified (anucleated) epithelial cells; estrus, cornified cells; metestrus, leukocytes, and cornified and nucleated epithelial cells; and diestrus, mostly leucocytes (Caligioni, 2009). All mice were actively cycling at the time of experiment, as confirmed by the characteristic changes in vaginal cytology during the 5 d preceding the kainic acid injection.

### Ago-Immunoprecipitation and qRT-PCR

For Ago2 immunoprecipitations (IPs), tissue was lysed in lysis buffer (50 mm Tris, pH 7.5, 40 mm NaCl, 1 mm EDTA, 0.5% Triton X-100, 50 mm NaF, 10 mm sodium pyrophosphate, and 10 mm sodium β-glycerol phosphate, containing proteinase and RNase inhibitors) followed by centrifugation at 15,000 × *g* and 4°C for 15 min. Protein content of the supernatant was determined using a Bradford assay (BIO-RAD). Twenty micrograms of the lysate were kept as an “input sample” at 4°C during the IP. Two hundred micrograms of hippocampal or cortical lysate were mixed with 4 μg of Ago2 antibody or mouse normal IgG as a control and were incubated for 4 h at 4°C on an end-over-end rotator. Then, the antibody–lysate mixture was incubated for 2 h at 4°C on an end-over-end rotator with G-protein-coupled agarose beads (Sigma-Aldrich). Beads were washed six times with wash buffer (20 mm Tris, pH 7.5, 150 mm NaCl, 5 mm MgCl2, and 1% NP-40, containing proteinase and RNase inhibitors), three times briefly, and three times with 10 min incubation on the rotator at 4°C. Then, RNA from IP and the input samples was extracted with TRIzol following the manufacturer protocol (Thermo Fisher Scientific). RNA from the IP and the input samples was dissolved in 18 and 36 μl water, respectively. The High Capacity RNA-to-cDNA kit (Thermo Fisher Scientific) was used for cDNA synthesis from mRNA using 9 μl of the RNA solution, and the qScript miRNA cDNA Synthesis Kit (Quanta BioSciences) or the miRCURY LNA miRNA PCR Starter Kit (Qiagen) configured for miR-324-5p and miR-191 were used to generate cDNA from miRNA using 7 μl of the RNA solution (diluted 1:60 for the miRCURY LNA miRNA PCR Kit). Target mRNAs or microRNAs were quantified using the SYBR green qRT-PCR Kit (BIO-RAD) on a QuantStudio 3 Real-Time PCR System (Thermo Fisher Scientific). Relative changes in expression were determined using the comparative cycle threshold method (2^−ΔCT^). The ΔCT was either the difference between IP and input samples (for Ago IPs), or the difference between Kv4.2 and GAPDH, or miR-324-5p and RU19 or miR-191, respectively (for total mRNA or miRNA). Because of the complex nature of this experiment, not all collected brain samples were successfully used for all experiments, and resulting *n* values are indicated in each figure. If mRNA or miRNA did not show enrichment in Ago2 IPs compared with IgG controls, experiments were excluded. If qRT-PCR duplicates differed by >0.6 CTs, they were excluded. IPs and/or qRT-PCRs were repeated as needed and possible. If several usable results for one sample were obtained, averages were calculated for this sample and used for the overall analyses. If any values were excluded as statistical outliers, this is indicated in the figure legend.

### Western blotting and quantification

For Kv4.2 protein quantification, tissue lysates were separated by SDS-PAGE, proteins were transferred electrophoretically to polyvinylidene fluoride membranes, and Kv4.2 and β3-tubulin as a loading control were detected by Western blot analysis. Briefly, after blocking in 5% milk or BSA, membranes were incubated with primary antibodies at 4°C overnight (Kv4.2, 1:2000; β3-tubulin, 1:500,000; both in 5% BSA). Species-specific horseradish peroxidase-coupled secondary antibodies were incubated for 1–2 h in 5% BSA at room temperature (1:2000), and the signal was detected using Pierce ECL Western Blotting Substrate (Thermo Fisher Scientific) and visualized using a ChemiDoc MP Imager from Bio-Rad Laboratories or autoradiography films (BioBlue-Lite Western Blot Film, Alkali Scientific) and a standard chemical developer. Kv4.2 detection was performed first, followed by stripping of the Western blot membrane using Restore Western Blot Stripping Buffer from Thermo Fisher Scientific, and detection of β3-tubulin. Specific bands were quantified densitometrically by experimenters who were blind to the condition using either Image Lab version 6.1.0 (BIO-RAD), or NIH ImageJ software (for films). Equal amounts of protein as determined by Bradford assays were loaded in duplicate, and signal intensities of Kv4.2-specific bands were normalized to β3-tubulin on the same blot. Equal amounts of hippocampal lysate from Kv4.2 knock-out mice collected during an unrelated study were run on each gel to confirm the Kv4.2-specific band.

### Quantification of progesterone and 17β-estradiol in plasma

Progesterone and 17β-estradiol in mouse plasma were quantified using commercially available ELISA kits [Progesterone (rat/mouse) ELISA kits from DRG International and Lifespan Bioscience; Mouse/Rat Estradiol ELISA kits from Calbiotech and Abcam, respectively]. Samples were analyzed in duplicate following manufacturer instructions. The total amounts of progesterone and 17β-estradiol as well as progesterone/17β-estradiol ratios were determined. For total progesterone levels, one mouse was removed as a statistical outlier using the Rout method. For 17β-estradiol levels, two mice were removed as statistical outliers.

### Experimental design and statistical analyses

The goal of this study was to assess whether female sex alters the regulation and therapeutic potential of targeting of miR-324-5p-mediated silencing of Kv4.2 in a mouse model of epilepsy. We used at least six mice per condition from four or more different litters for each experimental group as indicated in the subsection Mice. Statistical analyses were performed using GraphPad Prism version 9.2.0. Datasets were tested for normality using the Shapiro–Wilk test, and the appropriate parametric or nonparametric tests were used. Two-way ANOVA was used for analyses of data with two factors. Correlations were analyzed by computing the Pearson’s correlation coefficient *r*. Significance level was set to α = 0.05, and *p* < 0.05 was considered to be significant. Mice excluded because of technical failure are indicated under the respective methods. Statistical outliers were identified and removed using the Rout method, and the number of these statistical outliers (if any) are indicated in the figure legends. Individual data points in addition to the mean ± SEM are shown for comparisons of two or more groups. Correlations are presented as scatter plots with trend lines and 95% confidence intervals. Statistical tests used and their results as well as sample sizes are indicated in the figure legends. The *p*, *F*, *R*^2^, and *r* values were rounded to the second or third position after the decimal point.

## Results

### MiR-324-5p silencing in response to seizures is reduced in female mice

It has been shown previously that kainic acid-induced seizures lead to increased association of miR-324-5p with the RISC, suggestive of its increased activity, as well as increased miRNA-induced silencing of Kv4.2 mRNA and reduced protein levels of Kv4.2 in male mice (Gross et al., 2016). To test whether Kv4.2 is regulated similarly in female mice, we quantified protein expression and miRNA-induced silencing of Kv4.2 in response to seizures in female mice. Western blot analysis of hippocampal tissue from female C57BL6/J mice 90 min after kainic acid administration showed significantly reduced Kv4.2 protein expression [Fig. 1*B* (timeline shown in Fig. 1*A*)] and no change in Kv4.2 mRNA levels (Fig. 1*C*), as was similarly observed in male mice (Gross et al., 2016); however, in contrast to previous results in male mice, the association of Kv4.2 mRNA with Ago2 (i.e., with the RISC) was not significantly increased after seizure (Fig. 1*D*). Surprisingly, the association of miR-324-5p with Ago2 was significantly reduced (Fig. 1*E*), which is the opposite of what was observed in male mice (Gross et al., 2016). Similar to male mice, miR-324-5p total levels were unchanged after kainic acid in female mice (Fig. 1*F*). In the cortex, no significant changes in any of these parameters were detected (Extended Data Fig. 1-1). Notably, miR-324-5p association with Ago2 in the cortex showed a trend toward being reduced after kainic acid treatment, as similarly observed in the hippocampus (*p* = 0.06; Extended Data Fig. 1-1*E*). These results suggest that miRNA-induced silencing in response to seizures is regulated differently in female mice compared with male mice.

### MiR-324-5p inhibition does not consistently reduce seizure susceptibility in female mice

**Figure 2.**
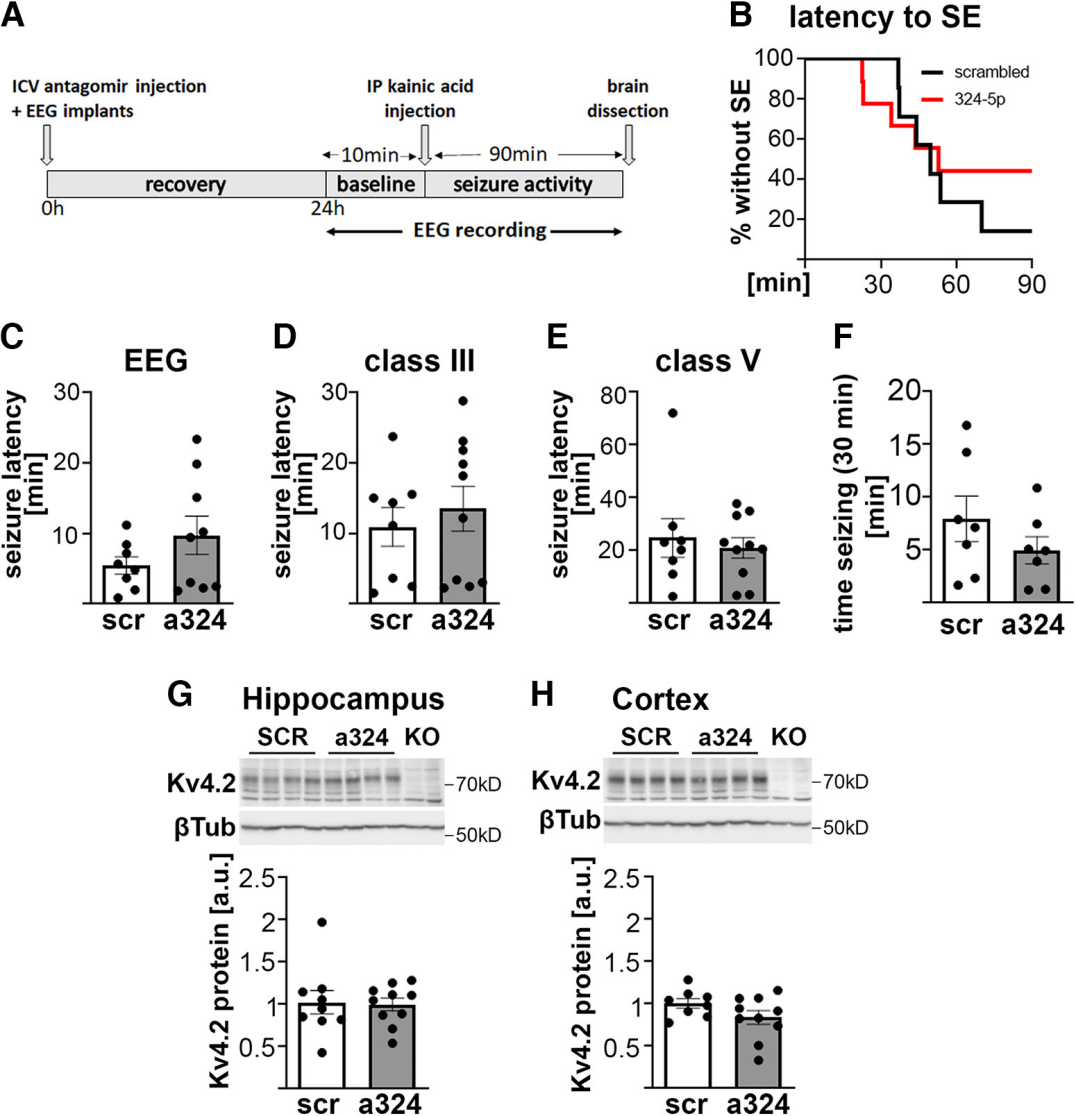
MiR-324-5p inhibition does not consistently increase latency to kainic acid-mediated seizure onset or reduce seizure severity in female mice. ***A***, Illustration depicting the experimental timeline. ***B***, Latency to and occurrence of status epilepticus are not altered in female mice by miR-324-5p antagomir treatment (log-rank (Cox–Mantel) test, *p* = 0.53; Fisher’s exact test, *p* = 0.31; *n*(SCR) = 7, *n*(a324) = 9). ***C–E***, Latency to seizure onset as determined by EEG (***C***) and by behavior (***D***, Racine class 3 seizures; ***E***, Racine class 5 seizures) is not significantly changed by antagomir treatment (***C***, unpaired *t* test, *p* = 0.191; *n*(SCR) = 8, *n*(a324) = 9; ***D***, unpaired *t* test, *p* = 0.563; *n*(SCR) = 8, *n*(a324) = 10; ***E***, Mann–Whitney test, *p* = 0.965; *n*(SCR) = 8, *n*(a324) = 10). ***F***, Time spent seizing in the first 30 min after kainic acid is on average reduced after miR-324-5p inhibition but not significantly different compared with scrambled antagomir-treated mice (unpaired *t* test, *p* = 0.26; *n* = 7). ***G***, ***H***, Hippocampal (***G***) and cortical (***H***) Kv4.2 protein levels after kainic acid-induced seizures are not significantly changed by miR-324-5p antagomirs compared with scrambled antagomirs (***G***, unpaired *t* test, *p* = 0.868; *n*(SCR) = 9, *n*(a324) = 10; ***H***, *p* = 0.13, unpaired *t* test; *n*(SCR) = 8, *n*(a324) = 10). One statistical outlier from the scrambled group in ***H*** was removed. Kv4.2 protein levels were normalized to β3-tubulin levels on the same blot. scr, scrambled antagomir; a324, anti-miR-324-5p antagomir. Bars and error bars represent the mean ± SEM. Additional EEG analyses, levels, and RISC association of Kv4.2 mRNA as well as correlation of biochemical measures with seizure severity are shown in Extended Data Figure 2-1.

It has previously been shown that in male mice, inhibiting miR-324-5p by intracerebroventricular injection of an antagomir delays onset to kainic acid-induced seizures and reduces seizure frequency in the pilocarpine model of temporal lobe epilepsy (Gross et al., 2016; Tiwari et al., 2019). Our results showing reduced association of miR-324-5p with the RISC after seizure in female mice (Fig. 1*E*) suggested that miR-324-5p inhibition may not be anticonvulsant in female mice. We, therefore, tested whether the miR-324-5p-specific antagomir delays kainic acid-induced seizure onset in female mice using a similar experimental approach used previously in male mice (Gross et al., 2016; Fig. 2*A*, timeline). MiR-324-5p inhibition did not significantly alter the latency to status epilepticus (Fig. 2*B*) or the probability to go into status epilepticus, although, on average, fewer anti-miR-324-5p-treated mice experienced status epilepticus (scr, 85.7%; anti-miR-324-5p. 55.6%; Fisher’s exact test, *p* = 0.31). MiR-324-5p-specific antagomirs did not consistently delay the onset of seizures identified by characteristic changes in EEG signal in adult female wild-type mice (Fig. 2*B*). To assess whether the antagomir changed the onset and severity of behavioral seizures, we also classified seizures according to a modified Racine scale (for details, see Materials and Methods), but likewise did not detect significant differences in the two treatment groups; however, overall, the female mice had a variable response (Fig. 2*C–E*). Last, we quantified the numbers and duration of seizures within the first 30 min after kainic acid injection. There were no significant differences in the time spent seizing (Fig. 2*F*), the number of seizures (Extended Data Fig. 2-1*A*), or the average duration of seizures (Extended Data Fig. 2-1*B*) during this time period. The previous studies in male mice showed that miR-324-5p inhibition increases Kv4.2 protein expression and prevents its seizure-induced downregulation (Gross et al., 2016). Western blot analyses of hippocampal and cortical lysates from female mice, however, did not show an increase in Kv4.2 protein compared with a scrambled control (Fig. 2*G*,*H*). In line with these findings, we also did not detect a decrease in Ago2 association of Kv4.2 mRNA after miR-324-5p antagomir; instead, the Ago2 association of Kv4.2 mRNA was increased after antagomir treatment in hippocampus, but not cortex (Extended Data Fig. 2-1*C*,*D*), further supporting the notion that miRNA-induced silencing of Kv4.2 is differentially regulated in male and female mice. No significant correlation of seizure latency or burden with Ago2 association of Kv4.2 mRNA (Extended Data Fig. 2-1*E*,*G*), Kv4.2 protein levels (Extended Data Fig. 2-1*F*,*H*), or Kv4.2 mRNA (Extended Data Fig. 2-1*I*,*J*) in hippocampus or cortex were observed.

10.1523/ENEURO.0047-22.2023.f2-1 Figure 2-1 MiR-324-5p inhibition does not change numbers or duration of seizures after kainic acid treatment in female mice, and miRNA-induced silencing of Kv4.2 does not correlate with seizure severity. ***A***, ***B***, Number (***A***) and duration (***B***) of seizures within the first 30 min after kainic acid injection are not significantly changed after treatment with miR-324-5p antagomirs compared with scrambled controls (unpaired *t* tests, *n* = 7; ***A***, *p* = 0.35; ***B***, *p* = 0.79). ***C***, ***D***, Association of Kv4.2 mRNA with the RISC is significantly increased after kainic acid-induced seizure in miR-324-5p antagomir-treated mice compared with scrambled antagomir-treated mice in the hippocampus (***C***), but no the cortex (***D***; unpaired *t* tests, *n* = 7; ***C***, *p* = 0.965). Bars and error bars represent the mean ± SEM. ***E–H***, No significant correlations of Kv4.2 mRNA RISC association (***E***, ***G***) and Kv4.2 protein (***F***, ***H***) in hippocampus or cortex with latency to seizure (***E***, ***F***) or time seizing (***G***, ***H***; Pearson’s correlations; ***E***: hippocampus: *r* = 0.46, *R*^2^ = 0.21, *p* = 0.11; *n* = 13; cortex: *r* = 0.41, *R*^2^ = 0.17, *p* = 0.14; *n* = 14; ***F***: hippocampus: *r* = –0.14, *R*^2^ = 0.02, *p* = 0.63; *n* = 14; cortex: *r* = 0.02, *R*^2^ < 0.001, *p* = 0.94; *n* = 15; ***G***: hippocampus: *r* = –0.34, *R*^2^ = 0.11, *p* = 0.31; *n* = 11; cortex: *r* = 0.16, *R*^2^ = 0.02, *p* = 0.65; *n* = 11; ***H***: hippocampus, *r* = 0.02, *R*^2^ < 0.01, *p* = 0.94; *n* = 11; cortex: *r* = 0.11, *R*^2^ = 0.01, *p* = 0.73; *n* = 12). ***I***, ***J***, Likewise, no significant correlations between hippocampal or cortical Kv4.2 mRNA and latency to seizure (***I***; Pearson’s correlations; hippocampus: *r* = –0.43, *R*^2^ = 0.18, *p* = 0.13; *n* = 14; cortex: *r* = 0.42, *R*^2^ = 0.42, *p* = 0.14; *n* = 13) or time seizing (***J***; Pearson’s correlations; hippocampus: *r* = 0.08, *R*^2^ < 0.01, *p* = 0.80; *n* = 12; cortex: *r* = –0.30, *R*^2^ = 0.41, *p* = 0.37; *n* = 11). Dashed lines indicate 95% confidence intervals. MiR-324-5p antagomir-treated mice are represented as red dots, and scrambled antagomir-treated mice as black dots. Pearson’s correlation statistics are also shown in the graphs in ***E–J***. Sample sizes in ***E–J*** differ because in some groups, certain samples were lost or excluded due to experimental failure, or lack of EEG (see Materials and Methods). Additional EEG analyses, as well as Kv4.2 protein levels in hippocampus and cortex after antagomir and kainic acid treatment are shown in Figure 2. Download Figure 2-1, TIF file.

### MiR-324-5p inhibition does not consistently increase Kv4.2 expression or prevent Kv4.2 mRNA association with the RISC as observed in male mice

**Figure 3.**
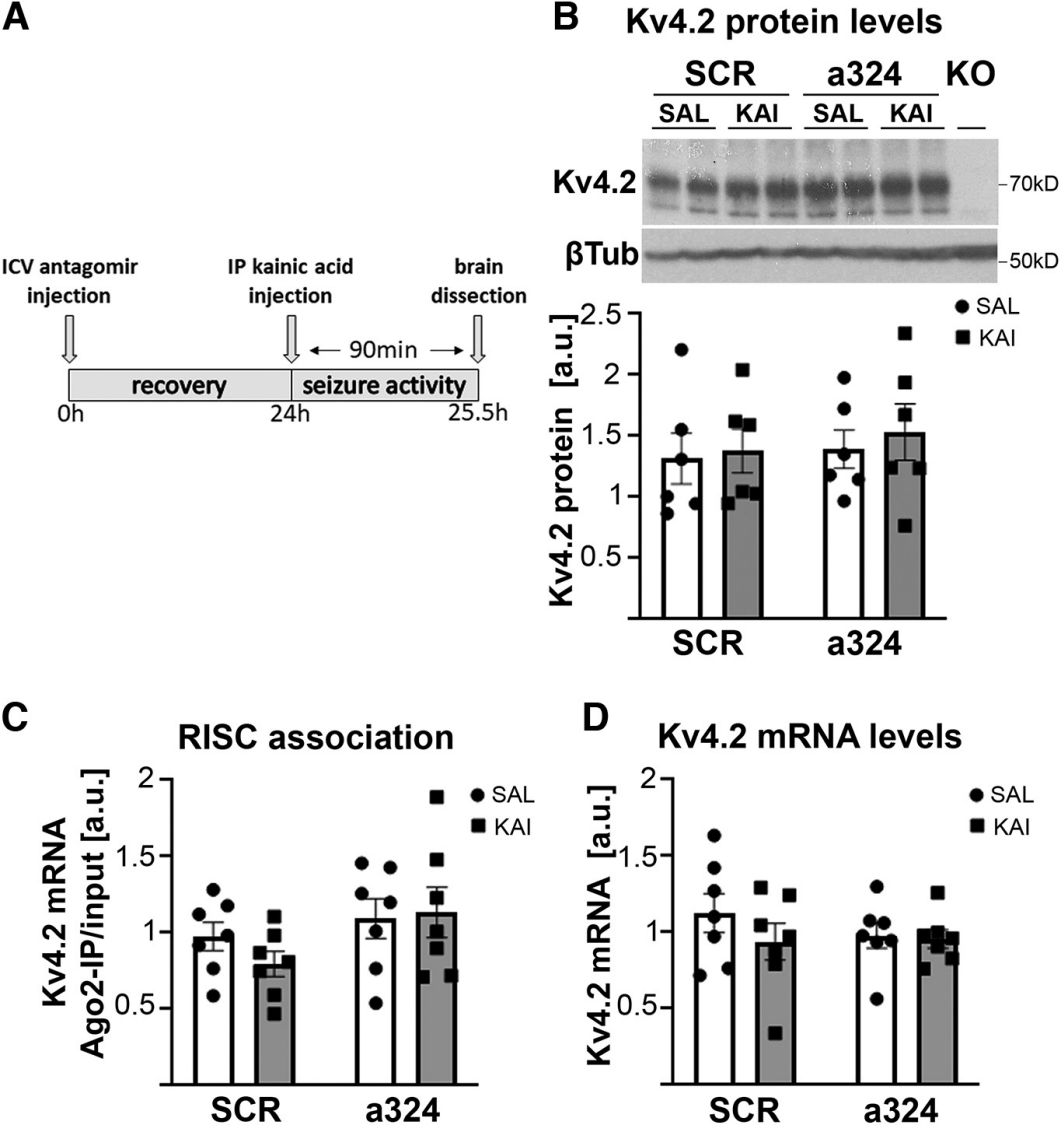
Stereotaxically delivered antagomirs combined with kainic acid-induced seizures do not change Kv4.2 protein levels, Kv4.2 mRNA levels, or RISC association of Kv4.2 mRNA in the hippocampus of female mice. ***A***, Illustration depicting the experimental timeline. MiR-324-5p-specific or scrambled antagomirs were stereotaxically intracerebroventricularly injected. Twenty-four hours later, 15 mg/kg kainic acid was intraperitoneally injected, and brains were dissected 90 min later. ***B***, Kv4.2 protein levels were not significantly changed in the hippocampus of female mice by antagomir or kainic acid treatment (two-way ANOVA; interaction: *F*_(1,20)_ = 0.04, *p* = 0.846; antagomir: *F*_(1,20)_ = 0.34, *p* = 0.564; kainic acid: *F*_(1,20)_ = 0.28, *p* = 0.61; *n* = 6/group). Kv4.2 protein levels were normalized to β3-tubulin levels on the same blot. ***C***, ***D***, Likewise, RISC association with (***C***) and levels of (***D***) Kv4.2 mRNA were not significantly changed in the hippocampus of female mice by antagomir or kainic acid treatment (two-way ANOVA; ***C***: interaction: *F*_(1,24)_ = 0.82, *p* = 0.37; effect of antagomir: *F*_(1,24)_ = 3.51, *p* = 0.073; effect of kainic acid: *F*_(1,24)_ = 0.33, *p* = 0.569; *n* = 7; ***D***: interaction: *F*_(1,24)_ = 0.64, *p* = 0.431; effect of antagomir: *F*_(1,24)_ = 0.4, *p* = 0.533; effect of kainic acid: *F*_(1,24)_ = 1.08, *p* = 0.308; *n* = 7). In ***B***, Kv4.2 protein was normalized to β-tubulin on the same blot, and in ***D***, Kv4.2 mRNA levels were normalized to Gapdh mRNA. scr, scrambled antagomir; a324, anti-miR-324-5p antagomir; a.u., arbitrary units. Bars and error bars represent the mean ± SEM. Analyses in cortical tissue are shown in Extended Data Figure 3-1. Analyses of control sham surgeries are shown in Extended Data Figure 3-2.

To test whether the observed lack of clear effects of the miR-324-5p antagomir on the expression of Kv4.2 in female mice was because of electrode implanting, we next analyzed a cohort of mice that just received stereotaxic injections of scrambled or miR-324-5p-specific antagomirs followed by kainic acid treatment 24 h later. Brain tissue was collected 90 min after kainic acid administration as was done before in male mice (Gross et al., 2016; Fig. 3*A*). We observed no significant changes in hippocampal Kv4.2 protein expression following antagomir injection and/or kainic acid treatment (Fig. 3*B*). Similarly, no effects on Kv4.2 mRNA association with the RISC (Fig. 3*C*) or total Kv4.2 mRNA levels (Fig. 3*D*) were detected in hippocampal lysates from these mice. Equivalent analyses in cortex also did not show consistent significant changes depending on antagomir injection and/or kainic acid treatment (Extended Data Fig. 3-1). Overall, values varied substantially between individual mice within one treatment group. We did not detect reduced hippocampal Kv4.2 protein levels after kainic acid treatment in the scrambled antagomir-injected mice (Fig. 3*B*), contrary to what we observed in mice that did not receive stereotaxic injections (Fig. 1*B*). We speculate that the surgery 24 h before kainic acid injection might have induced molecular changes specific to female mice (e.g., neuroinflammation, which is known to differ between male and female rodents; Osborne et al., 2018; Deal et al., 2022). These changes could have masked or altered kainic acid-induced effects and contributed to the observed variability. To test this hypothesis, we performed sham surgeries on female mice (intracerebroventricular injection of ACSF) followed by treatment with saline or kainic acid and tissue collection 24 h later (Extended Data Fig. 3-2). In these mice, hippocampal and cortical Kv4.2 protein levels were unchanged after seizure, as observed in scrambled antagomir-injected mice, supporting our hypothesis that the surgery or anesthesia induced molecular changes masking the effect of kainic acid. We replicated our findings in female mice that did not undergo surgery showing no change in Ago2 association of Kv4.2 mRNA after kainic acid treatment. A trend toward reduced miR-324-5p association after kainic acid was still observed in cortex, similar to the trend in nonsurgery mice (Extended Data Fig. 1-1), suggesting that female-specific regulation of miR-324-5p after seizure was partially intact. In summary, the results of the biochemical analyses, especially assessments of Kv4.2 protein levels, after antagomir injection thus have to be interpreted cautiously, as surgery-related changes might have masked antagomir-mediated effects.

10.1523/ENEURO.0047-22.2023.f3-1 Figure 3-1 Stereotaxically delivered antagomirs combined with kainic acid-induced seizures do not change Kv4.2 protein levels or RISC association of Kv4.2 mRNA in the cortex of female mice but change Kv4.2 mRNA levels. ***A***, Illustration depicting the experimental timeline. MiR-324-5p-specific or scrambled antagomirs were stereotaxically intracerebroventricularly injected. Twenty-four hours later, 15 mg/kg kainic acid was intraperitoneally injected and brains were dissected 90 min later. ***B***, Kv4.2 protein levels were not significantly changed in the cortex of female mice by antagomir or kainic acid treatment (2-way ANOVA; interaction: *F*_(1,20)_ = 0.04, *p* = 0.846; effect of antagomir: *F*_(1,20)_ = 0.34, *p* = 0.564; effect of kainic acid: *F*_(1,20)_ = 0.28, *p* = 0.61; *n* = 6/group). Kv4.2 protein levels were normalized to β3-tubulin levels on the same blot. ***C***, Likewise, RISC association of Kv4.2 mRNA was not significantly changed in the cortex of female mice by antagomir or kainic acid treatment (2-way ANOVA; interaction: *F*_(1,28)_ = 0.03, *p* = 0.873; effect of antagomir: *F*_(1,28)_ = 0.42, *p* = 0.521; effect of kainic acid: *F*_(1,28)_ = 2, *p* = 0.168; *n* = 8). ***D***, In contrast, Kv4.2 mRNA levels were significantly increased by seizure in the cortex of scrambled antagomir-injected female mice, but not in those of miR-324-5p-specific antagomir-injected female mice (2-way ANOVA with Dunnett’s multiple-comparison tests; interaction: *F*_(1,27)_ = 16.4, *p = 0.004; effect pf antagomir: *F*_(1,27)_ = 4.02, *p* = 0.0.055; effect of kainic acid: *F*_(1,27)_ = 2, *p* = 0.168, *p = 0.0015; *n* = 8, except for a324/kA, *n* = 7). Kv4.2 mRNA levels were normalized to Gapdh mRNA. a.u., Arbitrary units. Bars and error bars represent the mean ± SEM. Analyses in hippocampal tissue are shown in Figure 3. Download Figure 3-1, TIF file.

10.1523/ENEURO.0047-22.2023.f3-2 Figure 3-2 Sham surgeries blunt the effect of kainic acid-induced seizures on Kv4.2 protein levels in female mice. ***A***, Illustration depicting the experimental timeline. ACSF (vehicle) was stereotaxically intracerebroventricularly injected. Twenty-four hours later, 15 mg/kg kainic acid was intraperitoneally injected, and brains were dissected 90 min later. ***B***, Kv4.2 protein levels were not significantly changed in the hippocampus or cortex of female mice by kainic acid treatment 24 h after a sham surgery (hippocampus: unpaired *t* test, *p* = 0.413; *n* = 8; cortex: Mann–Whitney test, *p* = 0.593; *n* = 8). Kv4.2 protein levels were normalized to β3-tubulin levels on the same blot. Example blots shown on top. ***C***, Likewise, RISC association of Kv4.2 mRNA was not significantly changed in the hippocampus or cortex of sham female mice by kainic acid treatment (unpaired *t* tests: *p*(hippocampus) = 0.598, *p*(cortex) = 0.460; *n* = 8). ***D***, Kv4.2 mRNA levels were unchanged in hippocampus or cortex of sham intracerebroventricularly injected female mice after kainic acid-induced seizures (unpaired *t* tests: *p*(hippocampus) = 0.932, *p*(cortex) = 0.633; *n* = 8). Kv4.2 mRNA levels were normalized to Gapdh mRNA. ***E***, RISC association of miR-324-5p was not significantly changed in the hippocampus or cortex of sham female mice by kainic acid treatment (unpaired *t* tests: *p*(hippocampus) = 0.714, *n*(SAL) = 7, *n*(KAI) = 8; *p*(cortex) = 0.076; *n* = 8). ***F***, MiR-324-5p levels were unchanged in hippocampus of sham intracerebroventricularly injected female mice after kainic acid-induced seizures, but significantly reduced in the cortex (unpaired *t* tests; hippocampus: *p* = 0.176; *n*(SAL) = 7, *n*(KAI) = 8; one statistical outlier removed from the saline group; cortex: unpaired *t* test: *p* = 0.050, *n* = 8). MiR-324-5p levels were normalized to miR-191. a.u., Arbitrary units. Bars and error bars represent the mean ± SEM. Analyses of antagomir-injected mice in hippocampal and cortical tissue are shown in Figure 3 and Extended Data Figure 3-1, respectively. Download Figure 3-2, TIF file.

### MicroRNA-induced silencing of Kv4.2 is correlated with plasma levels of progesterone and estradiol

**Figure 4.**
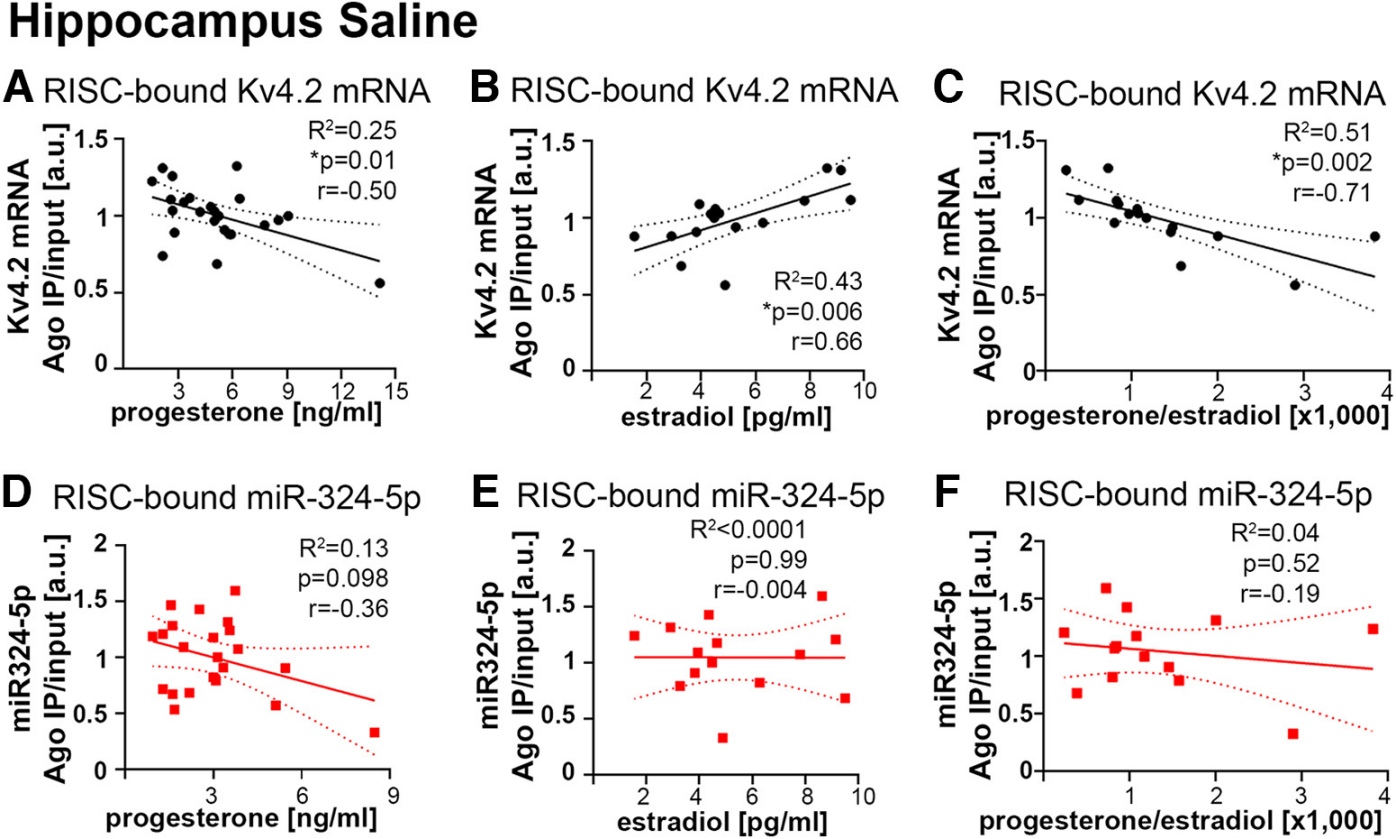
MicroRNA-induced silencing of Kv4.2 in the hippocampus is correlated with plasma levels of progesterone and 17β-estradiol. ***A–C***, Kv4.2 mRNA association with the RISC in hippocampal lysates from female mice is negatively correlated with plasma levels of progesterone (***A***; Pearson’s correlation: *r* = −0.50, *R*^2^ = 0.25, *p = 0.012; *n* = 24), positively correlated with plasma levels of 17β-estradiol (***B***; Pearson’s correlation: *r* = 0.66, *R*^2^ = 0.43, *p = 0.006; *n* = 16), and negatively correlated with progesterone/17β-estradiol ratios (***C***; Pearson’s correlation: *r* = −0.71, *R*^2^ = 0.51, *p = 0.002; *n* = 16). ***D–F***, By contrast, RISC association of the Kv4.2-targeting miRNA miR-324-5p only shows a trend toward a negative correlation with progesterone (***D***; Pearson’s correlation: *r* = −0.36, *R*^2^ = 0.13, *p* = 0.098; *n* = 22) and no correlation with 17β-estradiol (***E***; Pearson’s correlation: *r* = −0.004, *R*^2^ = 0.00,001, *p* = 1.00; *n* = 14) or progesterone/17β-estradiol ratios (***F***; Pearson’s correlation: *r* = −0.19, *R*^2^ = 0.04, *p* = 0.52; *n* = 14). Dashed lines indicate 95% confidence intervals. Pearson’s correlation statistics are also shown in the figure. Absence of significant correlations among progesterone, 17β-estradiol, or progesterone/17β-estradiol ratios and two other potassium channels, Kv7.2 and Kv7.3, is shown in Extended Data Figure 4-1.

**Figure 5.**
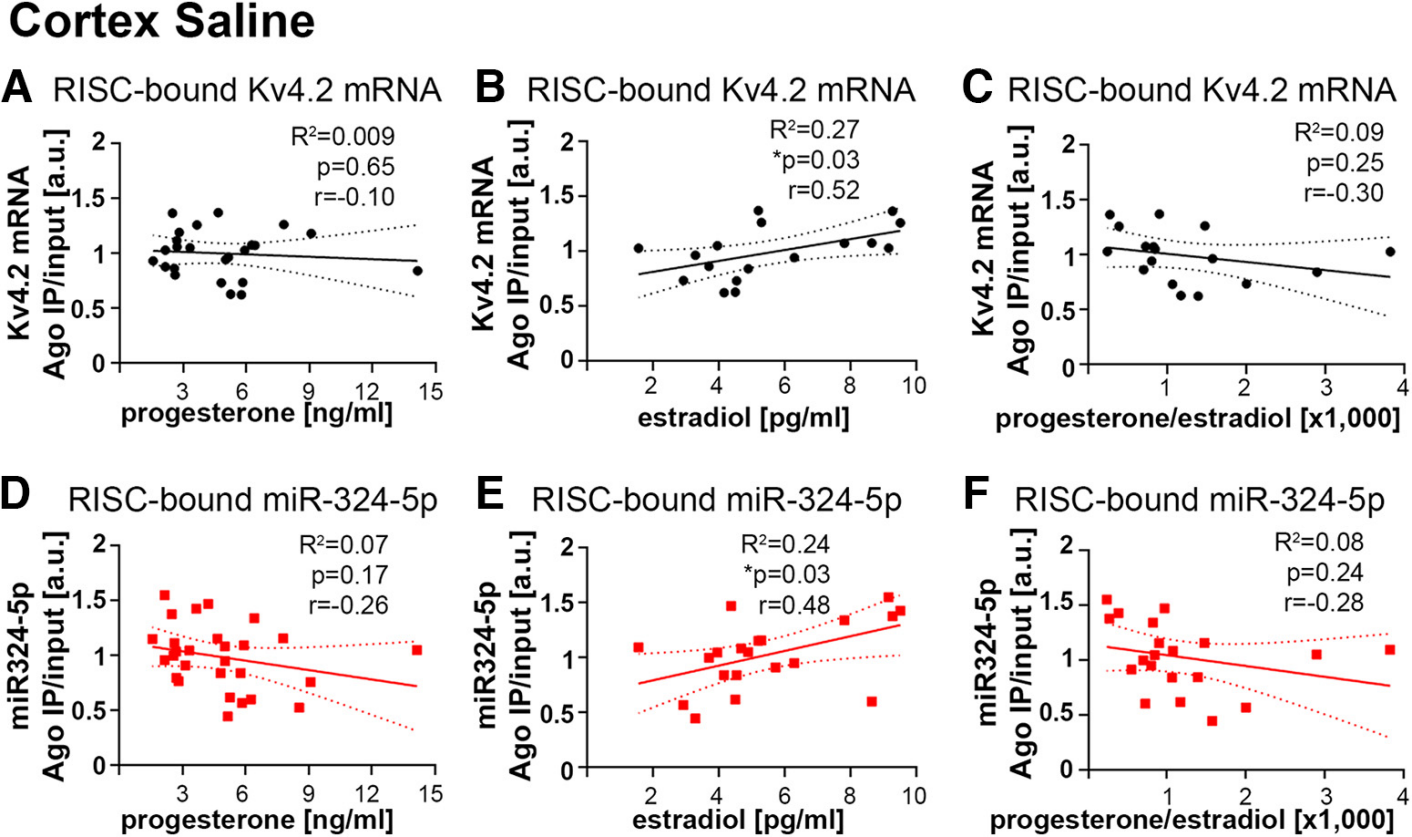
MicroRNA-induced silencing of Kv4.2 in the cortex is correlated with plasma levels of progesterone and 17β-estradiol. ***A–C***, There is no significant correlation of Kv4.2 mRNA association with the RISC in cortical lysates from female mice with plasma levels of progesterone (***A***; Pearson’s correlation: *r* = −0.10, *R*^2^ = 0.01, *p* = 0.65; *n* = 24) or progesterone/17β-estradiol ratios (***C***; Pearson’s correlation: *r* = −0.30, *R*^2^ = 0.09, *p* = 0.248; *n* = 17); however, Kv4.2 mRNA association with Ago2 is positively correlated with plasma levels of 17β-estradiol (***B***; Pearson’s correlation: *r* = 0.52, *R*^2^ = 0.27, *p = 0.034; *n* = 17) in cortical lysates, similarly to what was observed in hippocampus. ***D–F***, Similarly as Kv4.2 mRNA, RISC association of the Kv4.2-targeting miRNA miR-324-5p is not correlated with progesterone (***D***; Pearson’s correlation: *r* = −0.26, *R*^2^ = 0.07, *p* = 0.176; *n* = 28) and progesterone/17β-estradiol ratios in the cortex (***F***; Pearson’s correlation: *r* = −0.28, *R*^2^ = 0.08, *p* = 0.240; *n* = 20). Like Kv4.2 mRNA, miR-324-5p mRNA association with Ago2 is positively correlated with 17β-estradiol (***E***; Pearson’s correlation: *r* = 0.48, *R*^2^ = 0.24, *p = 0.030; *n* = 20). Dashed lines indicate 95% confidence intervals. Pearson’s correlation statistics are also shown in the figure.

Given the variability of the observed effects of antagomirs and kainic acid on both seizure onset and miRNA-induced silencing of Kv4.2 in female mice, we speculated that estrous cycle-associated hormonal fluctuations alter miRNA-induced silencing of Kv4.2. To test this hypothesis, we used ELISA to quantify plasma levels of progesterone and 17β-estradiol of the female mice analyzed in Figure 1 (saline and kainic acid groups, no antagomir treatment) using blood collected at the end of the experiment. 17β-Estradiol is the main active form of estrogens and is believed to mediate most functions in the brain (Sheppard et al., 2019); we thus used 17β-estradiol as a proxy for estrogens. Because of previous results that seizures alter miRNA-induced silencing of Kv4.2 in male mice (Gross et al., 2016), we first focused on saline-treated mice. Progesterone and 17β-estradiol concentrations varied between mice (progesterone, 1.6–14.1 ng/ml; 17β-estradiol, 1.5–9.5 pg/ml). We then analyzed whether progesterone or 17β-estradiol levels correlated with miRNA-induced silencing of Kv4.2 in hippocampus or cortex. In the hippocampus, Kv4.2 mRNA association with Ago2 (i.e., the RISC) was negatively correlated with progesterone levels (Fig. 4*A*) and was positively correlated with 17β-estradiol levels (Fig. 4*B*). MiR-324-5p association with Ago2 showed a trend of negative correlation with progesterone levels (Fig. 4*D*) but no correlation with 17β-estradiol levels (Fig. 4*E*). To account for differences in blood collection, which could affect absolute hormone concentration, we also performed similar analyses using ratios of progesterone to 17β-estradiol. Consistent with the results for progesterone and 17β-estradiol, we observed a significant negative correlation between progesterone/17β-estradiol ratios and the Kv4.2 mRNA association with Ago2 (Fig. 4*C*), but not for the miR-324-5p association with Ago2 (Fig. 4*F*). In the cortex, neither miR-324-5p nor Kv4.2 association with the RISC was correlated with progesterone levels and progesterone/17β estradiol ratios (Fig. 5*A*,*C*,*D*,*F*). By contrast, RISC association with both was positively correlated with 17β-estradiol (Fig. 5*B*,*E*). Kv4.2 protein and mRNA levels measured by Western blot and qRT-PCR, respectively, were not significantly correlated with hormonal levels in hippocampus or cortex (Table 1).

10.1523/ENEURO.0047-22.2023.f4-1 Figure 4-1 RISC association of the voltage-gated potassium channels Kv7.2 and Kv7.3 in the hippocampus are not correlated with plasma levels of progesterone or 17β-estradiol. ***A–C***, Kv7.2 mRNA association with the RISC in hippocampal lysates from female mice is not significantly correlated with plasma levels of progesterone (***A***; Pearson’s correlation: *r* = 0.22, *R*^2^ = 0.05, *p* = 0.54; *n* = 10), 17β-estradiol (***B***; Pearson’s correlation: *r* = 0.41, *R*^2^ = 0.17, *p* = 0.24; *n* = 10), and progesterone/17β-estradiol ratios (***C***; Pearson’s correlation: *r* = –0.03, *R*^2^ = 0.001, *p* = 0.93; *n* = 10). ***D–F***, Likewise, RISC association of Kv7.3 mRNA is not significantly correlated with progesterone (***D***; Pearson’s correlation: *r* = 0.24, *R*^2^ = 0.06, *p* = 0.54; *n* = 9), 17β-estradiol (***E***; Pearson’s correlation: *r* = 0.48, *R*^2^ = 0.23, *p* = 0.19; *n* = 9), or progesterone/17β-estradiol ratios (***F***; Pearson’s correlation: *r* = –0.07, *R*^2^ = 0.006, *p* = 0.85; *n* = 9). Dashed lines indicate 95% confidence intervals. Pearson’s correlation statistics are also shown in the figure. Corresponding analyses of Kv4.2 are shown in Figure 4. Download Figure 4-1, TIF file.

**Table 1:** No significant correlations between Kv4.2 protein and mRNA levels and 17β-estradiol or progesterone in hippocampus or cortex of 6- to 8-week-old saline-treated female mice

		17β- Estradiol	Progesterone	Proestrus/ estrous
Hippocampus	Protein	*r* = −0.20	*r* = −0.09	*r* = 0.27
		*R*^2^ = 0.04	*R*^2^ = 0.00	*R*^2^ = 0.08
		*p* = 0.38	*p* = 0.6	*p* = 0.2
		*n* = 21	*n* = 29	*n* = 21
	mRNA	*r* = −0.12	*r* = 0.13	*r* = −0.06
		*R*^2^ = 0.01	*R*^2^ = 0.02	*R*^2^ = 0.004
		*p* = 0.64	*p* = 0.55	*p* = 0.81
		*n* = 17	*n* = 25	*n* = 17
Cortex	Protein	*r* = 0.03	*r* = 0.16	*r* = −0.02
		*R*^2^ = 0.00	*R*^2^ = 0.03	*R*^2^ = 0.00
		*p* = 0.89	*p* = 0.42	*p* = 0.92
		*n* = 20	*n* = 28	*n* = 20
	mRNA	*r* = −0.18	*r* = −0.08	*r* = −0.05
		*R*^2^ = 0.03	*R*^2^ = 0.006	*R*^2^ = 0.003
		*p* = 0.48	*p* = 0.71	*p* = 0.84
		*n* = 17	*n* = 24	*n* = 17

We next tested whether a correlation between sex hormones and mRNA association with the RISC could also be observed for other voltage-gated potassium channels involved in epilepsy, using Kcnq2 and Kcnq3 (called Kv7.2 and Kv7.3, respectively) as examples. Our analyses in hippocampal tissue did not show significant correlations of progesterone or 17β-estradiol with those two channels, suggesting that the effect is not generalized (Extended Data Fig. 4-1).

### Kainic acid-induced seizures alter correlations between hormone levels and Kv4.2 mRNA association with the RISC

**Figure 6.**
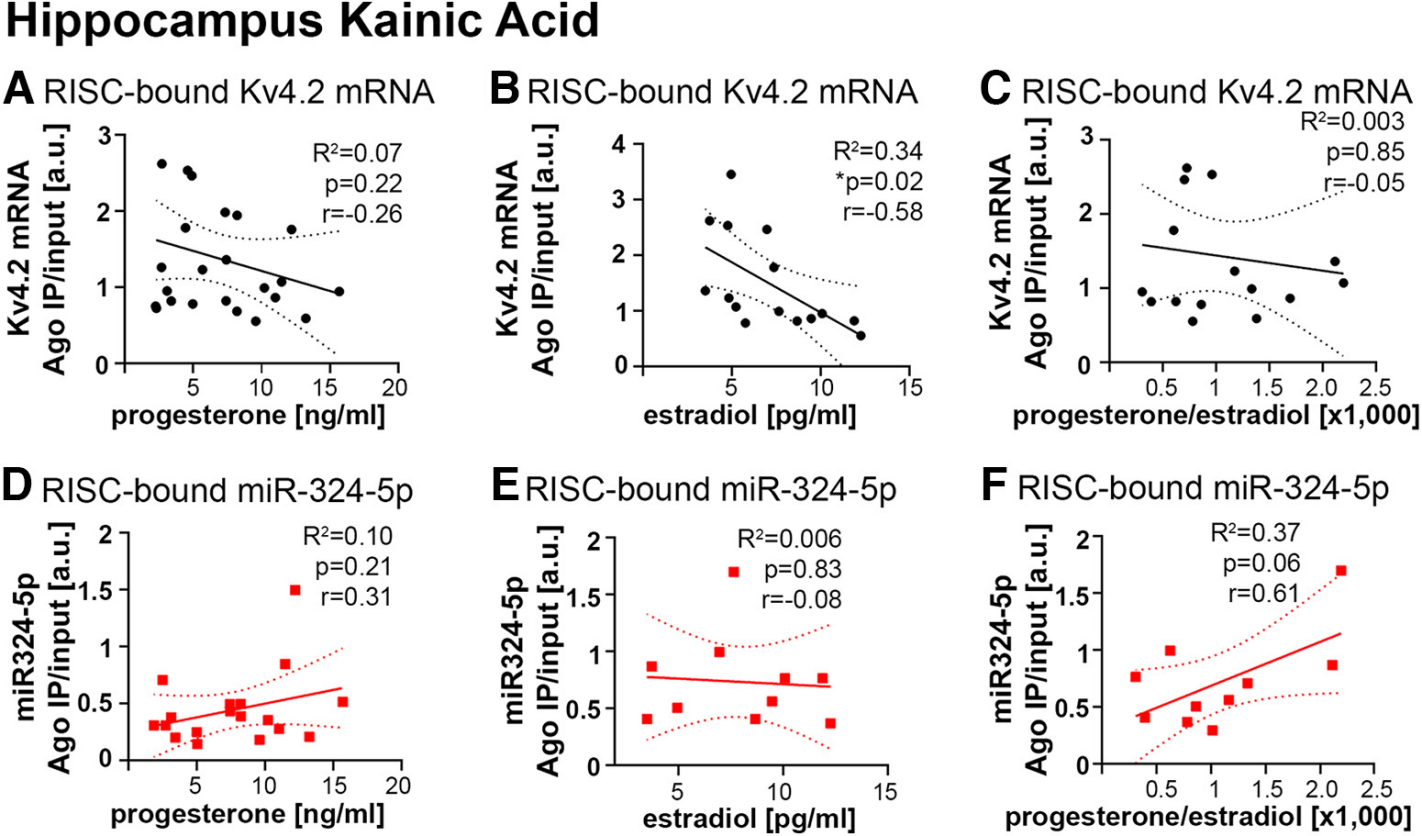
Treatment with kainic acid alters correlations of miRNA-induced silencing of Kv4.2 in the hippocampus with plasma levels of progesterone and 17β-estradiol. ***A–C***, Ninety minutes following kainic acid treatment, Kv4.2 mRNA association with the RISC in hippocampal lysates from female mice is not correlated with plasma levels of progesterone (***A***; Pearson’s correlation: *r* = −0.26, *R*^2^ = 0.067, *p* = 0.223; *n* = 24), is negatively correlated with plasma levels of 17β-estradiol (***B***; Pearson’s correlation: *r* = −0.58, *R*^2^ = 0.34, *p = 0.023; *n* = 15) and is not significantly correlated with progesterone/17β-estradiol ratios (***C***; Pearson’s correlation: *r* = −0.05, *R*^2^ = 0.003, *p* = 0.85; *n* = 15). ***D–F***, RISC association of the Kv4.2-targeting miRNA miR-324-5p is not significantly correlated with progesterone (***D***; Pearson’s correlation: *r* = 0.31, *R*^2^ = 0.10, *p* = 0.210; *n* = 18) but shows a trend toward positive correlation with progesterone/17β-estradiol ratios (***F***; Pearson’s correlation: *r* = 0.61, *R*^2^ = 0.37, *p* = 0.06; *n* = 10). No significant correlation with 17β-estradiol was detected (***E***; Pearson’s correlation: *r* = −0.08, *R*^2^ = 0.006, *p* = 0.83; *n* = 10). Dashed lines indicate 95% confidence intervals. Pearson’s correlation statistics are also shown in the figure. Analyses in the cortex are shown in Extended Data Figure 6-1.

Epilepsy can lead to disruption of the estrous cycle, and seizure burden fluctuates with the estrous cycle (Li et al., 2020). In addition, miRNA-induced silencing of Kv4.2 mRNA and Kv4.2 protein levels can be altered by acute seizures and in epilepsy (Fig. 1; Gross et al., 2016; Tiwari et al., 2019). We thus tested whether neuronal hyperactivity and seizures induced by kainic acid affected the correlations of Kv4.2 and miR-324-5p RISC association with hormonal levels. Indeed, kainic acid treatment altered correlations of Kv4.2 and miR-324-5p RISC loading with progesterone and 17β-estradiol in hippocampus and cortex (Fig. 6, Extended Data Fig. 6-1). Most correlations were lost or even reversed (positive correlation of RISC-bound Kv4.2 with estradiol after saline, negative correlation after kainic acid; Figs. 4*B*, 6*B*). Moreover, miR-324-5p association with RISC showed a trend toward positive correlation with progesterone/17β-estradiol ratios after kainic acid in the hippocampus (Fig. 6*F*), whereas no significant correlation was observed in saline-treated mice (Fig. 4*F*). It is important to note that the sample size for 17β-estradiol correlation analyses of miR-324-5p Ago2 association was rather small, therefore, results have to be interpreted with caution. In cortical tissue, all correlations were lost after kainic acid treatment (Extended Data Fig. 6-1). Overall, the variability of the data was higher after kainic acid treatment compared with saline treatment. As in the saline-treated mice, progesterone and 17β-estradiol varied between mice (progesterone, 1.8–15.7 ng/ml; 17β-estradiol, 3.5–12.3 pg/ml). Although, on average, both progesterone and 17β-estradiol levels were higher in kainic acid-treated mice, no significant differences between saline and kainic acid were detected (progesterone: SAL = 4.89 ± 0.49 ng/ml; KAI = 6.50 ± 0.68 ng/ml; Mann–Whitney test, *p* = 0.15; 17β-estradiol: SAL = 5.38 ± 0.49 pg/ml; KAI = 6.57 ± 0.63 pg/ml; Mann–Whitney test, *p* = 0.17; data not shown).

10.1523/ENEURO.0047-22.2023.f6-1 Figure 6-1 In the cortex, treatment with kainic acid abolishes all significant correlations of microRNA-induced silencing of Kv4.2 with plasma levels of progesterone and 17β-estradiol. ***A–C***, Ninety minutes following kainic acid treatment, Kv4.2 mRNA association with the RISC in cortical lysates from female mice is not significantly correlated with plasma levels of progesterone (***A***; Pearson’s correlation: *r* = –0.05, *R*^2^ = 0.003, *p* = 0.80; *n* = 27), 17β-estradiol (***B***; Pearson’s correlation: *r* = 0.02, *R*^2^ = 0.0004, *p* = 0.94; *n* = 16), and progesterone/17β-estradiol ratios (***C***; Pearson’s correlation: *r* = –0.20, *R*^2^ = 0.04, *p* = 0.47; *n* = 16). ***D–F***, Likewise, no significant correlations between RISC association of miR-324-5p with plasma progesterone (***D***; Pearson’s correlation: *r* = –0.20, *R*^2^ = 0.04, *p* = 0.33; *n* = 25), 17β-estradiol was detected (***E***, Pearson’s correlation: *r* = 0.07, *R*^2^ = 0.005, *p* = 0.79; *n* = 16), and progesterone/17β-estradiol ratios (***F***; Pearson’s correlation: *r* = –0.11, *R*^2^ = 0.01, *p* = 0.70; *n* = 16) was observed. Dashed lines indicate 95% confidence intervals. Pearson’s correlation statistics are also shown in the figure. Analyses in hippocampal tissue are shown in Figure 6. Download Figure 6-1, TIF file.

**Figure 7.**
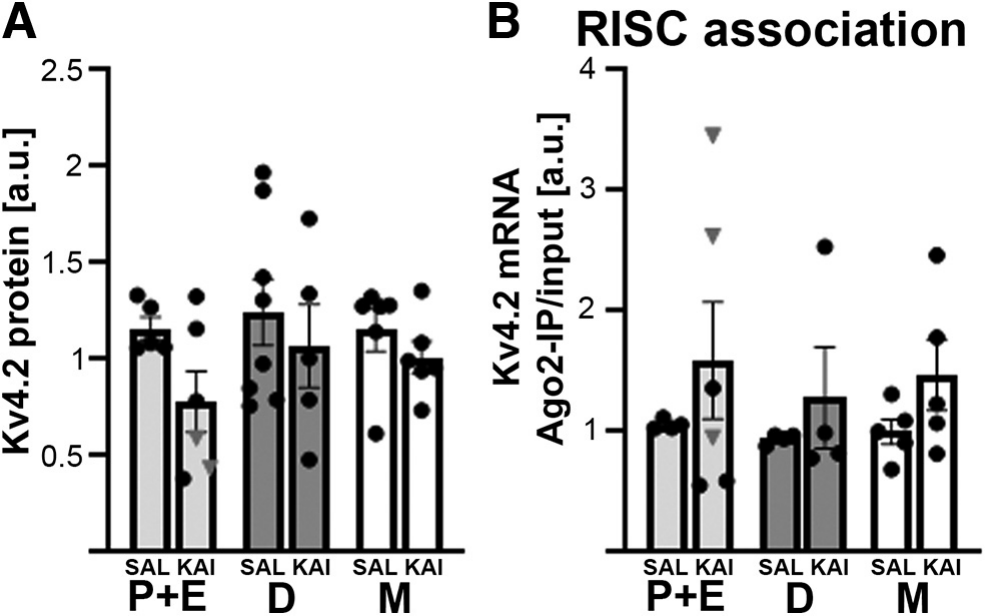
No significant differences in Kv4.2 protein expression or RISC association of Kv4.2 mRNA across estrous cycle stages and depending on kainic acid treatment in the hippocampus. ***A***, Kv4.2 protein levels in the hippocampus are not significantly different across estrous stages in female saline-treated or kainic acid-treated mice, and no significant interaction between the treatment stage and estrous stage was detected. Consistent with results shown in Figure 1, a trend toward reduced protein levels after kainic acid was observed (two-way ANOVA; interaction: *F*_(2,30)_ = 0.36, *p* = 0.70; effect of estrous stage: *F*_(2,30)_ = 0.77, *p* = 0.474; effect of kainic acid: *F*_(1,30)_ = 3.6, *p* = 0.07; *n*(proestrus+estrus saline) = 5, *n*(proestrus+estrus kainic acid) = 6, *n*(diestrus saline) = 8, *n*(diestrus kainic acid) = 5, *n*(metestrus saline) = 6, *n*(metestrus kainic) = 6). Kv4.2 was normalized to β3-tubulin signal on the same blot. ***B***, Kv4.2 mRNA association with the RISC in the hippocampus is not significantly different across estrous stages in female saline-treated or kainic acid-treated mice (two-way ANOVA; interaction: *F*_(2,22)_ = 0.04, *p* = 0.96; effect of estrous stage: *F*_(2,22)_ = 0.20, *p* = 0.824; effect of kainic acid: *F*_(1,22)_ = 2.7, *p* = 0.11, *n*(proestrus+estrus saline) = 4, *n*(proestrus+estrus kainic acid) = 6, *n*(diestrus saline) = 4, *n*(diestrus kainic acid) = 4, *n*(metestrus saline) = 5, *n*(metestrus kainic) = 5). P + E, Proestrus and estrous; D, diestrus; M, metestrus. No mice in proestrus were identified in the saline group. Mice in proestrus in the kainic group are indicated as gray triangles in the bar diagram. Mice used for this analysis are a subset of the mice analyzed in Figures 1, 4, and 6. Analyses of cortical tissue as well as of miR-324-5p association with the RISC are shown in Extended Data Figure 7-1.

To test whether Kv4.2 protein levels or RISC association of Kv4.2 mRNA and miR-324-5p differed between estrous cycle stages, we used vaginal cytology to group a subset of the female mice used for the correlation analyses into metestrus, diestrus, proestrus, and estrus. Vaginal swabs were taken on 5 consecutive days, including the day of the experiment, to confirm that all mice were cycling. Because of the low number of mice in proestrus (e.g., none in the saline group), proestrus and estrus were combined. We did not detect significant differences in Kv4.2 protein expression or RISC association of Kv4.2 mRNA in the hippocampus of saline-treated or kainic acid-treated mice across proestrus/estrus, metestrus, and diestrus (Fig. 7). No significant interactions between estrous cycle stage and kainic acid treatment and no main effects of estrous cycle stage were detected. Of note, in line with pairwise comparisons shown in Figure 1, a trend toward a main effect of treatment was detected for Kv4.2 protein levels (*p* = 0.067, *F*_(1,30)_ = 3.60), but not for Ago2 association of Kv4.2 mRNA (*p* = 0.114, *F*_(1,22)_ = 2.7) or miR-324-5p (*p* = 0.24, *F*_(1,14)_ = 1.5). Similarly, no significant interactions or main effects for Kv4.2 protein expression of Kv4.2 mRNA RISC association were detected in cortical tissue (Extended Data Fig. 7-1*A*,*B*), and there were no changes in miR-324-5p RISC association in hippocampus or cortex (Extended Data Fig. 7-1*C*,*D*). Low sample sizes for some conditions may have contributed to lack of significant effects. We examined vaginal cytology directly before saline or kainic acid injections and at the time of tissue collection (90 min later) and did not detect differences in cycle stage (data not shown).

10.1523/ENEURO.0047-22.2023.f7-1 Figure 7-1 No significant differences in miR-324-5p association with the RISC in hippocampus or cortex, and no changes in Kv4.2 protein expression and Kv4.2 mRNA association with the RISC in the cortex across estrous cycle stages and depending on kainic acid treatment. ***A***, Kv4.2 protein levels in the cortex not significantly different across estrous stages in female saline-treated or kainic acid-treated mice (two-way ANOVA; interaction: *F*_(2,29)_ = 1.4, *p* = 0.26; effect of estrous stage: *F*_(2,29)_ = 1.2, *p* = 0.307; effect of kainic acid: *F*_(1,29)_ = 0.06, *p* = 0.807; *n*(proestrus+estrus saline) = 5, *n*(proestrus+estrus kainic acid) = 7, *n*(diestrus saline) = 8, *n*(diestrus kainic acid) = 5, *n*(metestrus saline) = 5, and *n*(metestrus kainic) = 5). Kv4.2 was normalized to β3-tubulin signal on the same blot. ***B***, Kv4.2 mRNA association with the RISC in the cortex is not significantly different across estrous stages in female saline-treated or kainic acid-treated mice (two-way ANOVA; interaction: *F*_(2,24)_ = 1.8, *p* = 0.19; effect of estrous stage: *F*_(2,24)_ = 1.5, *p* = 0.23; effect of kainic acid: *F*_(1,24)_ = 0.8, *p* = 0.37; *n*(proestrus+estrus saline) = 2, *n*(proestrus+estrus kainic acid) = 7, *n*(diestrus saline) = 7, *n*(diestrus kainic acid) = 5, *n*(metestrus saline) = 5, and *n*(metestrus kainic) = 4). ***C***, MiR-324-5p association with the RISC in the hippocampus is not significantly different across estrous stages in female saline-treated or kainic acid-treated mice (two-way ANOVA; interaction: *F*_(2,14)_ = 1, *p* = 0.39; effect of estrous stage: *F*_(2,14)_ = 0.58, *p* = 0.57; effect of kainic acid: *F*_(1,14)_ = 1.5, *p* = 0.24; *n*(proestrus+estrus saline) = 3, *n*(proestrus+estrus kainic acid) = 4, *n*(diestrus saline) = 3, *n*(diestrus kainic acid) = 3, *n*(metestrus saline) = 5, and *n*(metestrus kainic) = 2). ***D***, MiR-324-5p association with the RISC in the cortex is not significantly different across estrous stages in female saline-treated or kainic acid-treated mice (two-way ANOVA; interaction: *F*_(2,27)_ = 1.4, *p* = 0.272; effect of estrous stage: *F*_(2,27)_ = 0.29, *p* = 0.75; effect of kainic acid: *F*_(1,27)_ = 1.5, *p* = 0.22; *n*(proestrus+estrus saline) = 5, *n*(proestrus+estrus kainic acid) = 6, *n*(diestrus saline) = 7, *n*(diestrus kainic acid) = 4, *n*(metestrus saline) = 5, and *n*(metestrus kainic) = 6). +E, Proestrus and estrous; D, diestrus; M, metestrus. No mice in proestrus were identified in the saline group. Mice in proestrus in the kainic group are indicated as gray triangles in the bar diagram. The mice used for this analysis are a subset of the mice analyzed in Figures 1, 4, and 6. Analyses of Kv4.2 protein and Kv4.2 mRNA association with the RISC are shown in Figure 7. Download Figure 7-1, TIF file.

### Ovariectomized mice do not show changes in miR-324-5p activity or miRNA-mediated silencing of Kv4.2 following kainic acid-induced seizures

**Figure 8.**
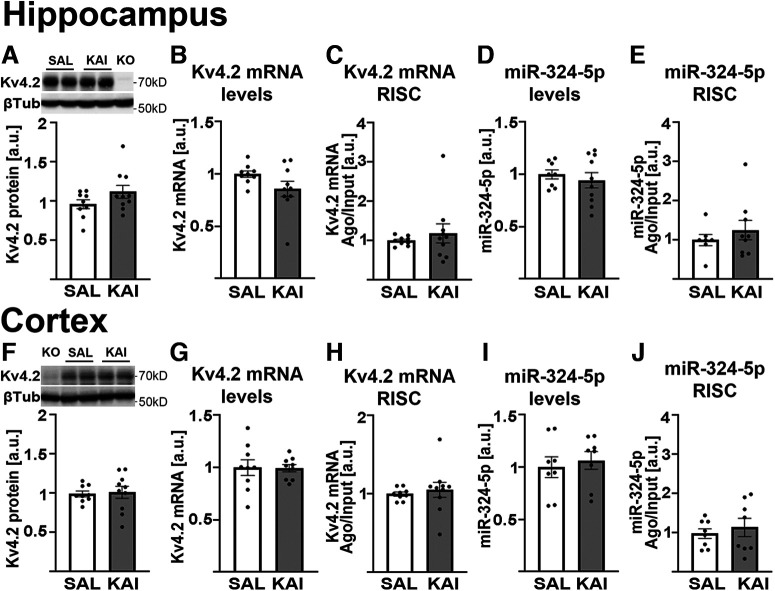
No significant differences in Kv4.2 expression or miRNA-induced silencing after kainic acid treatment in ovariectomized female mice. ***A–J***, Mice were ovariectomized, injected intraperitoneally with kainic acid after 14 d of recovery, and hippocampal (***A–E***) and cortical (***F–J***) tissue was collected 90 min later (Fig. 1*A*, timeline). ***A***, ***F***, Kv4.2 protein levels were not significantly different following kainic acid treatment in hippocampus (***A***; unpaired *t* test, *p* = 0.135; *n*(SAL) = 9, *n*(KAI) = 10) or cortex (***F***; unpaired *t* test, *p* = 0.821; *n*(SAL) = 9, *n*(KAI) = 10) in ovariectomized mice. ***B***, ***G***, Likewise, no changes in Kv4.2 mRNA levels were observed in hippocampus (***B***; unpaired *t* test, *p* = 0.101; *n*(SAL) = 9, *n*(KAI) = 10) or cortex (***G***; unpaired *t* test, *p* = 0.935; *n*(SAL) = 9, *n*(KAI) = 9; one statistical outlier removed). ***C***, ***H***, No changes in Kv4.2 mRNA association with the RISC in hippocampus (***C***; Mann–Whitney test, *p* = 0.604, *n*(SAL) = 9, *n*(KAI) = 10) or cortex (***H***; Mann–Whitney test, *p* = 0.447; *n*(SAL) = 9, *n*(KAI) = 10) were detected. ***D***, ***I***, Likewise, miR-324-5p levels in hippocampus (***D***; unpaired *t* test, *p* = 0.545; *n* = 9) or cortex (***I***; unpaired *t* test, *p* = 0.623; *n* = 8) were unchanged after kainic acid in ovariectomized mice. ***E***, ***J***, RISC association of miR-324-5p was also not different after kainic acid in hippocampus (***E***; Mann–Whitney test, *p* = 0.757; *n*(SAL) = 7, *n*(KAI) = 9) or cortex (***J***; unpaired *t* test, *p* = 0.556; *n* = 8) of ovariectomized mice. Kv4.2 protein was normalized to β-tubulin on the same blot, Kv4.2 mRNA levels were normalized to Gapdh mRNA, and miR-324-5p total levels were normalized to RU19 or miR-91 in the same samples. SAL, saline; KAI, kainic acid; a.u., arbitrary units. Bars and error bars represent the mean ± SEM.

We speculated that, if the sex differences in miRNA-induced silencing after seizure in mice were solely mediated by female gonadal hormones, then ovariectomized female mice, in which gonadal hormone production is disrupted, would phenocopy male mice. To test this hypothesis, we next treated a group of ovariectomized female mice with kainic acid and quantified Kv4.2 protein and mRNA levels, miR-324-5p levels, as well as Kv4.2 mRNA and miR-324-5p association with Ago2 in the hippocampus or cortex. In contrast to male and intact female mice, we did not detect changes in any of these measures after kainic acid-induced seizures 2 weeks following ovariectomy (Fig. 8).

## Discussion

It is known that neurologic diseases vary between men and women; however, studies investigating underlying molecular mechanisms and how those affect the efficacy of approved or suggested treatment strategies are still relatively scarce [e.g., a recent editorial discussing this subject (The Lancet Neurology, 2019)]. Our study contributes to understanding of the molecular mechanisms underlying sex differences in neuronal function by showing that miRNA-induced silencing of potassium channels in the brain changes with peripheral levels of female sex hormones, which may affect the efficacy of potential future miRNA-based treatment strategies in males and females. Using the voltage-gated potassium channel Kv4.2 and miR-324-5p as examples, we show that plasma levels of 17β-estradiol and progesterone in sexually mature female mice are correlated with the degree of silencing of Kv4.2 and the silencing activity of miR-324-5p in cortex and hippocampus. As a likely consequence, an antagomir to miR-324-5p, which reduces seizure susceptibility in male mice partially through increasing Kv4.2 (Gross et al., 2016), does not consistently reduce seizure susceptibility in a female mouse model. These findings are important as they suggest that the efficacy of potential miRNA-targeting therapeutics may differ between men and women and could be affected by hormonal changes during the menstrual cycle. Overall, this work corroborates the importance of studying molecular mechanisms in both males and females, and emphasizes the need of including female subjects in preclinical studies identifying brain disease mechanisms and treatments.

Female sex and female sex hormones alter neuronal excitability, seizure susceptibility, and epilepsy development (Christian et al., 2020). Catamenial epilepsy, for example, is a form of epilepsy in which seizure burden varies across the menstrual cycle (Frank and Tyson, 2020). These changes are recapitulated in female mouse models of epilepsy (Maguire et al., 2005). In line with a pivotal role of female sex hormones and the estrous cycle in neuronal excitability, we observed that the association of the proconvulsant miR-324-5p and Kv4.2 with the RISC in the brain are correlated with plasma levels of progesterone and/or 17β-estradiol. Kv4.2 association with the RISC, a measure of the degree of miRNA-induced silencing of Kv4.2, is negatively correlated with progesterone and positively correlated with 17β-estradiol in the hippocampus. This suggests that Kv4.2 is less silenced and more functionally expressed in the hippocampus with higher progesterone levels, but more silenced and less functionally expressed with higher estradiol levels. As Kv4.2 is hyperpolarizing and increasing its expression reduces neuronal excitability (Tiwari et al., 2020), this finding is in line with an anticonvulsant effect of progesterone and a proconvulsant effect of estrogens, as shown in previous studies (Sato and Woolley, 2016; Joshi et al., 2018). Our results suggest a novel molecular mechanism involving microRNAs and Kv4.2 that contributes to the differential effects of the female sex hormones progesterone and 17β-estradiol on neuronal excitability.

Interestingly, Kv4.2 mRNA association with the RISC is negatively correlated with progesterone plasma levels only in the hippocampus (*r* = −0.5, *p* = 0.01), but not the cortex (*r* = −0.1, *p* = 0.65), whereas a positive correlation with 17β-estradiol is detected in both brain regions. This suggests differences in the correlation of miRNA-induced silencing with gonadal hormones in peripheral blood depending on the brain region. In this context, it is of interest that clinical trials with allopregnanolone (the active neurosteroid derived from progesterone) in women with epilepsy have not shown the expected strong seizure-suppressing effect (Herzog et al., 2012). Recent studies have suggested that the regulation of neuronal excitability by progesterone and its derivatives is more complicated than originally thought and involves both proexcitatory and antiexcitatory effects (Kapur and Joshi, 2021). Our results showing brain region specificity of miRNA-induced silencing correlating with female gonadal hormones may add another layer of complexity to the actions of progesterone in the brain. While they support an antiexcitatory effect of progesterone, as the anticonvulsant Kv4.2 is less silenced with increasing progesterone levels in hippocampus, the lack of an effect in cortex may hint to differential regulation of miRNA-induced silencing of Kv4.2 by progesterone depending on the brain region, which may affect how allopregnanolone regulates seizure susceptibility.

In line with brain region specificity of the interaction of gonadal hormone plasma levels with miRNA-induced silencing activity, miR-324-5p association with the RISC is only significantly correlated with 17β-estradiol in the cortex (*r* = 0.48, *p* = 0.03), but not the hippocampus (*r* = −0.004, *p* = 0.99). As miR-324-5p is a proconvulsant miRNA, inhibition of which reduces seizure susceptibility, the observed positive correlation of cortical miR-324-5p activity with 17β-estradiol supports proexcitatory actions of estrogens. These results also suggest, however, that cortical and hippocampal activity of miR-324-5p is regulated differentially in females, which is expected to alter how miR-324-5p-specific antagomirs affect the hippocampal and cortical target mRNAs of miR-324-5p and, thus, excitability in females. Future studies with larger sample sizes are required to fully understand the potential brain region-specific regulation of miRNA-induced silencing of Kv4.2 by female sex hormones. The results reported in this article were obtained from an acute seizure model, which limits interpretation regarding chronic epilepsy. Future studies are needed in mouse models of catamenial epilepsy to evaluate whether similar mechanisms modulate seizure burden in chronic epilepsy.

Maybe not surprisingly, kainic acid treatment altered the correlations between miR-324-5p-induced silencing of Kv4.2 and sex hormones. While there was still a negative correlation of Ago2-associated Kv4.2 mRNA with progesterone in the hippocampus after kainic acid, it was not significant anymore and the correlation with 17β-estradiol was reversed. Seizure activity increases Kv4.2 mRNA association with the RISC in male mice (Gross et al., 2016). Our experiments did not show significant changes in Kv4.2 mRNA RISC association after kainic acid treatment in females; however, RISC association was on average increased, and more subtle changes could alter the correlation with sex hormones. Moreover, it could be that the timing of changes in Kv4.2 silencing after seizure is different in male and female mice, which could be studied in time course experiments in the future. After kainic acid treatment, miR-324-5p activity showed a trend toward positive correlation with progesterone/17β-estradiol ratios (*r* = 0.61, *p* = 0.06), but was not correlated with 17β-estradiol, suggesting that kainic acid affects miR-324-5p differently compared with its target Kv4.2. MRNAs are recruited to the RISC by pairing with microRNAs. Our findings therefore also suggest that other microRNAs, apart from miR-324-5p, regulate Kv4.2 after seizures in females. Investigations to address these questions are needed to further delineate the mechanisms underlying the interplay between female sex hormones, neuronal hyperexcitation, and miRNA-induced silencing. A limitation of this study is that it is unclear whether kainic acid treatment affected progesterone and 17β-estradiol levels. To the best of our knowledge, there are no studies analyzing the acute effect of kainic acid on progesterone or 17β-estradiol levels in the plasma. We did not detect significant differences in these hormones in the saline group compared with the kainic acid group; however, both hormones were on average higher after kainic acid treatment. Whether or not this is a true effect and could have contributed to the results we observed, will have to be further studied with larger sample sizes in the future.

A previous study has shown that δGABA_A_ receptor expression in the brain varies across different estrous cycle stages, which alters tonic inhibition and neuronal excitability (Maguire et al., 2005). This suggests that different estrous stages are defined by discrete molecular changes in neurons; however, we did not detect significant differences in RISC association or expression of Kv4.2 and miR-324-5p between estrous cycle stages despite the significant correlations between sex hormone levels and miRNA-induced silencing of Kv4.2. Progesterone and 17β-estradiol levels change within one estrous cycle stage (Walmer et al., 1992). For example, 17β-estradiol/progesterone ratios are low during the beginning of diestrus but start to rise toward the end of this stage. Thus, if miRNA-induced silencing of Kv4.2 is mainly associated with hormonal levels, then separation into four estrous cycle stages based on vaginal cytology would not show significant differences, as suggested by our data. Overall, these data support the hypothesis that progesterone and 17β-estradiol hormonal status, but not discrete estrous stages, are associated with miR-324-5p activity and miRNA-mediated silencing of Kv4.2.

As a first attempt to study the influence of female gonadal hormones on sex-dependent differences in kainic acid-induced regulation of miRNA-induced silencing, we assessed ovariectomized mice. We did not detect any significant effects on Kv4.2 protein and mRNA levels, or miRNA-mediated silencing, or miR-324-5p levels and activity. These results support an important role of gonadal hormones in reduced association of miR-324-5p with the RISC after seizure observed in female mice. They also suggest that sex differences independent of gonadal hormone production alter kainic acid-dependent regulation of miRNA-induced silencing of Kv4.2. The fact that no reduction in Kv4.2 protein could be detected in ovariectomized mice following seizure may indicate a role of gonadal hormones in Kv4.2 downregulation in female mice and suggest that mechanisms leading to reduced Kv4.2 protein levels after kainic acid differ in male and intact female mice. Therefore, we conclude that while gonadal hormones play a role in the regulation of miR-324-5p activity and Kv4.2 protein levels by kainic acid in female mice, they do not fully account for the observed sex differences. Of note, there are differences in male and female brain development, and progesterone and 17β-estradiol can be locally synthesized in the brain, which both could be a source of gonadal hormone-independent changes (Schumacher et al., 2004; Rossetti et al., 2016). Here, we focused on peripheral plasma levels of these hormones, but future studies are needed to assess the contribution of peripherally and locally produced 17β-estradiol and progesterone. To stay within the same age range of mice used for previous experiments, we studied short-term effects of ovariectomy (2 weeks). More work is needed to study longer-term effects of gonadal hormone disruption in female mice. To keep experimental conditions consistent between intact and ovariectomized female mice, we did not implant the mice with EEG electrodes. A limitation of this experiment is, therefore, that we were not able to assess seizure severity in the ovariectomized mice, which could have affected Kv4.2 protein levels and miRNA-induced silencing. Previous studies have shown that ovariectomy in rats leads to faster progression to status epilepticus after pilocarpine injection compared with intact female rats (Scharfman et al., 2005) and that ovariectomized mice have a lower threshold to pentylenetetrazole-induced seizures compared with mice in diestrus (Riazi et al., 2004). This suggests that the ovariectomized mice had earlier and more severe seizures than some of the intact female mice. A previous study in male mice (Tiwari et al., 2019) showed that Kv4.2 protein was not reduced 3 weeks after pilocarpine-induced status epilepticus, a time when only a few mice experience spontaneous seizures, but was significantly reduced 5 weeks after status epilepticus, when all mice experience spontaneous seizures, suggesting that, in males, reduction in Kv4.2 protein levels is exacerbated with increased seizure burden, the opposite of what we have observed here. Moreover, in a small cohort of intact female mice, we did not detect correlations between latency to seizure onset or time seizing and Kv4.2 protein levels (Extended Data Fig. 2-1*F*,*H*). Lack of reduction in Kv4.2 protein after kainic acid in ovariectomized female mice is thus not likely to be caused by differences in seizure severity.

We did not show clear changes in Kv4.2 protein expression with varying hormone levels. This might be because of the method used to assess Kv4.2 protein levels: Western blot are semiquantitative and not suitable to reliably detect subtle changes. Moreover, we did not investigate whether the changes in Kv4.2 silencing are correlated with changes in A-type currents, and thus have functional consequences. A change in A-type currents because of altered silencing of Kv4.2 with changes in female sex hormones is likely for the following two reasons: it has been previously shown that the inhibition of miR-324-5p-induced silencing of Kv4.2 in mice increases A-type currents in hippocampal slices (Tiwari et al., 2019) and that estrogens regulate A-type currents in primary hippocampal and immortalized GT1-7 neurons (Farkas et al., 2007; Zhang et al., 2015). Additional studies are needed to thoroughly test the effects of estrogens and progesterone on Kv4.2-mediated A-type potassium currents.

There are several limitations to our study. As alluded to above, sample sizes were relatively small for some experiments, and thus effects must be interpreted with caution. Moreover, the kainic acid dose that we used yielded seizures with inconsistent severity in female mice, which could have contributed to the variability in the data. Finally, the surgeries that were required to implant electrodes and inject antagomirs affected how Kv4.2 and miR-324-5p were regulated by seizures in females. The underlying causes that led to these differences after surgeries are unknown. Previous studies in male mice did not suggest that the surgeries changed miRNA-induced silencing of Kv4.2 (Gross et al., 2016; Tiwari et al., 2019). Anesthesia has been shown to lead to changes in progesterone in young women (Soules et al., 1980). Thus, a possible explanation is that the surgeries altered the estrous cycle and hormonal status of females. Moreover, it has been reported that neuroinflammation and activation of microglia differs in male and female mice (Osborne et al., 2018; Deal et al., 2022), which might have contributed to surgery-related mechanisms changing Kv4.2 regulation in female mice. In line with the assumption of a confounding effect of surgeries, no reduction in protein after kainic acid treatment was observed in female mice that underwent sham surgeries. Thus, unspecific effects of surgery could have contributed to the lack of a consistent effect of intracerebroventricularly administered miR-324-5p antagomir on seizure susceptibility in female mice.

While our studies do not show whether there is a causal relationship between changes in sex hormone levels and miRNA-induced silencing, they strongly suggest that the activity of miR-324-5p and silencing of Kv4.2 mRNA are influenced by biological sex and sex hormones. Apart from investigating a potential causal relationship, it will also be interesting to assess whether the observed effects are specific to select microRNAs and their targets, or whether the miRNA-induced silencing complex in general is influenced by biological sex and sex hormones. Our work suggests that the efficacy of miR-324-5p antagomirs (and potentially other candidate microRNAs) changes with hormonal status. It will be important to further test this hypothesis and evaluate potential combination therapies manipulating miRNA activity and hormonal levels. Related to this, the effect of other female or male sex hormones on miRNA-induced silencing will have to be investigated. In summary, this work encourages future studies analyzing how female sex and estrous cycle affect miRNA-induced silencing and its value as a potential therapeutic target in epilepsy.

## Synthesis

Reviewing Editor: Christina Zelano, Northwestern University - Chicago Decisions are customarily a result of the Reviewing Editor and the peer reviewers coming together and discussing their recommendations until a consensus is reached. When revisions are invited, a fact-based synthesis statement explaining their decision and outlining what is needed to prepare a revision will be listed below. The following reviewer(s) agreed to reveal their identity: NONE.

The authors have made many improvements over the previous version of the manuscript, in particular the addition of more mice to improve statistical rigor, assessment of ovariectomized mice, and clarifications and edits to the text. The results continue to indicate an interesting relationship between Kv4.2 regulation and circulating progesterone/estradiol, and that this relationship is altered after acute seizures. However, there are still some remaining concerns about the analyses and lack of certain controls, as detailed below.

1. EEG analysis is limited to seizure latency. More extensive EEG analysis (e.g., time in epileptiform activity, number of seizures) would be much more informative. Moreover, even within the latency parameters there is quite a bit of variability. Correlation statistics should be incorporated to determine whether the biochemical data show a relationship with the seizure burden experienced by each mouse.

2. The additional data from OVX mice are very helpful and indeed show an important relationship between circulating ovarian hormones and Kv4.2 regulation in the brain. However, given that differences between ovary-intact and OVX mice in underlying seizure susceptibility have been documented, the authors should incorporate an analysis of the acute seizure burden in these groups. It is possible that the differences between OVX vs. intact females, and remaining differences between OVX females and males, reflect differences in the severity of seizures the groups experienced prior to tissue collection.

3. The authors suggest that changes induced by the surgeries explain the discrepancy between results for the control groups in Figures 1 and 3. Although this is a reasonable speculation, these experiments still appear rather problematic to interpret. If the surgical procedure creates such a significant confound that the controls cannot replicate a principal finding, how can the authors conclude what the effects of the antagomir are? At least with respect to the possibility of anesthesia creating a confound, this could be directly addressed by examining sham surgical controls.

4. The Significance Statement still includes reference to a “change with natural fluctuations of estrogens and progesterone” and the last sentence of the Introduction states “regulated by natural fluctuations in female sex hormones.” This phrasing is still too strong in relation to the data presented, which show individual values of these hormones for each mouse.

5. In Figure 7, data from proestrous and estrous mice are combined due to the small sample size. Given the increased level of variability in the kainic acid-injected group, it would be helpful to use different colors or icon shapes to indicate which data points are from proestrus and which are from estrus.

Minor:

1. Line 175: please state the time of lights-off, in relation to injections between 1-4 pm.

2. Line 422: there is a typo in the data for estradiol levels, which should be in pg/ml.
