# Supplementary figures and images for: Estradiol- and Progesterone-Associated Changes in microRNA-Induced Silencing and Reduced Antiseizure Efficacy of an Antagomir in Female Mice
Source: eNeuro. 2023 Jul 21;10(7):ENEURO.0047-22.2023. doi: 10.1523/ENEURO.0047-22.2023 (PMC10368146; doi:10.1523/ENEURO.0047-22.2023)

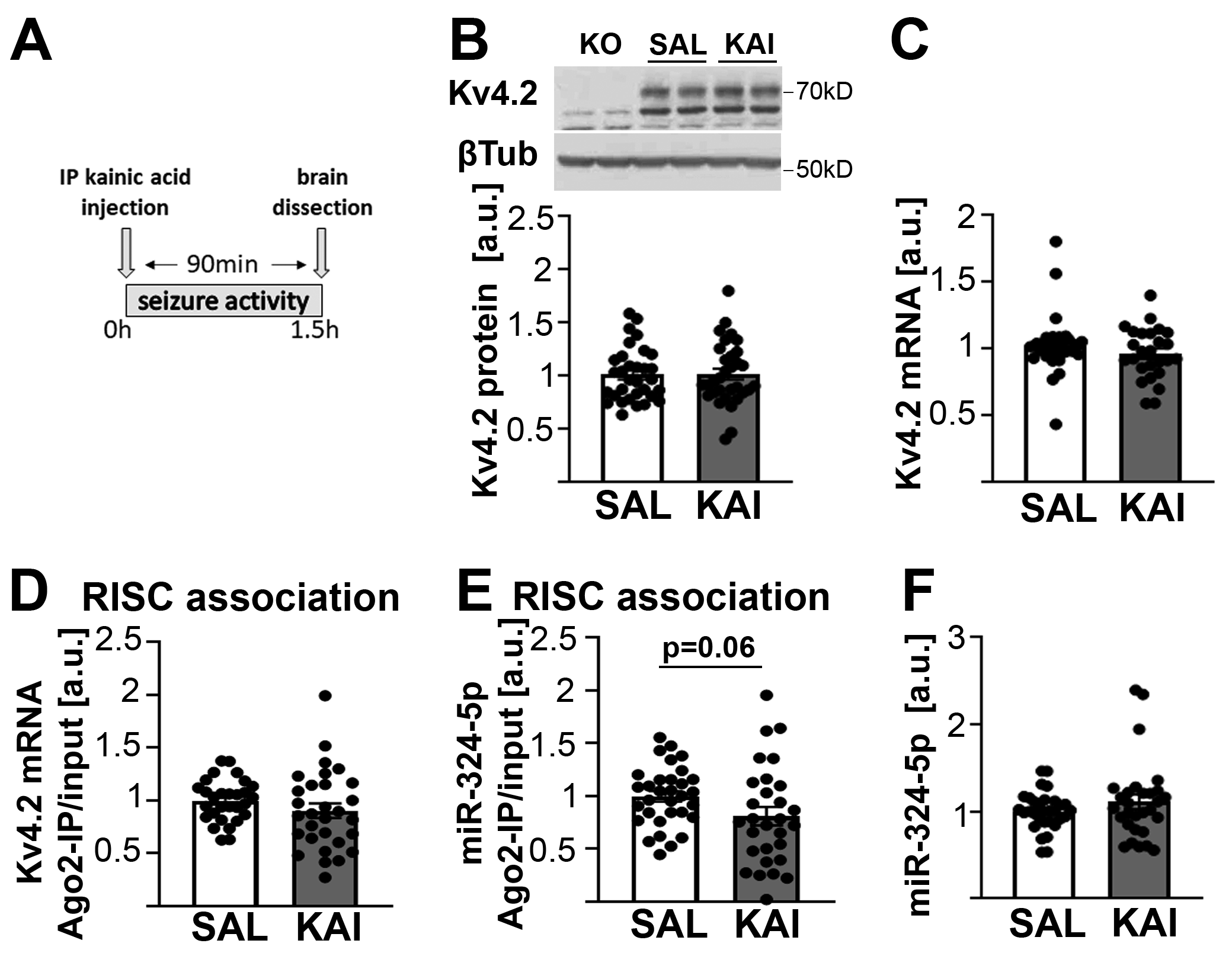

Supplement: Figure 1-1 — Kv4.2 protein levels and microRNA-induced silencing of Kv4.2 mRNA are unchanged in the cortex of female mice 90 min following kainic acid-induced seizure. A, Timeline of kainic acid injection and tissue harvest. B, C, Cortical Kv4.2 protein levels are unchanged in female mice 90 min following injection of 15 mg/kg, i.p., kainic acid (B; unpaired t test, p = 0.937, n = 32/group). Similarly, Kv4.2 mRNA levels are unchanged (C; unpaired t test, p = 0.372, n = 26; 3 statistical outliers removed from the saline group). Kv4.2 protein levels were normalized to β3-tubulin levels on the same blot, and Kv4.2 mRNA was normalized to Gapdh mRNA in the same samples. D, RISC association of Kv4.2 mRNA is not changed after kainic acid in the cortex of female mice (unpaired t test, p = 0.226; n(SAL) = 29, n(KAI) = 30; 1 statistical outlier removed from kainic acid group). E, F, Cortical RISC association of the Kv4.2-targeting microRNA miR-324-5p shows a trend towards being reduced in female mice following kainic acid (E; unpaired t test, p = 0.062; n(SAL) = 32, n(KAI) = 29), and miR-324-5p levels are unchanged (F; unpaired t test, p = 0.838, n(SAL) = 31, n(KAI) = 27; 2 statistical outliers removed from the kainic acid group). In F, miR-324-5p total levels were normalized to RU19 or miR-91 in the same samples. a.u., Arbitrary units. Bars and error bars represent the mean ± SEM. Analyses in hippocampal tissue are shown in Figure 1. Download Figure 1-1, TIF file. [file enu-eN-NWR-0047-22-s02.tif]

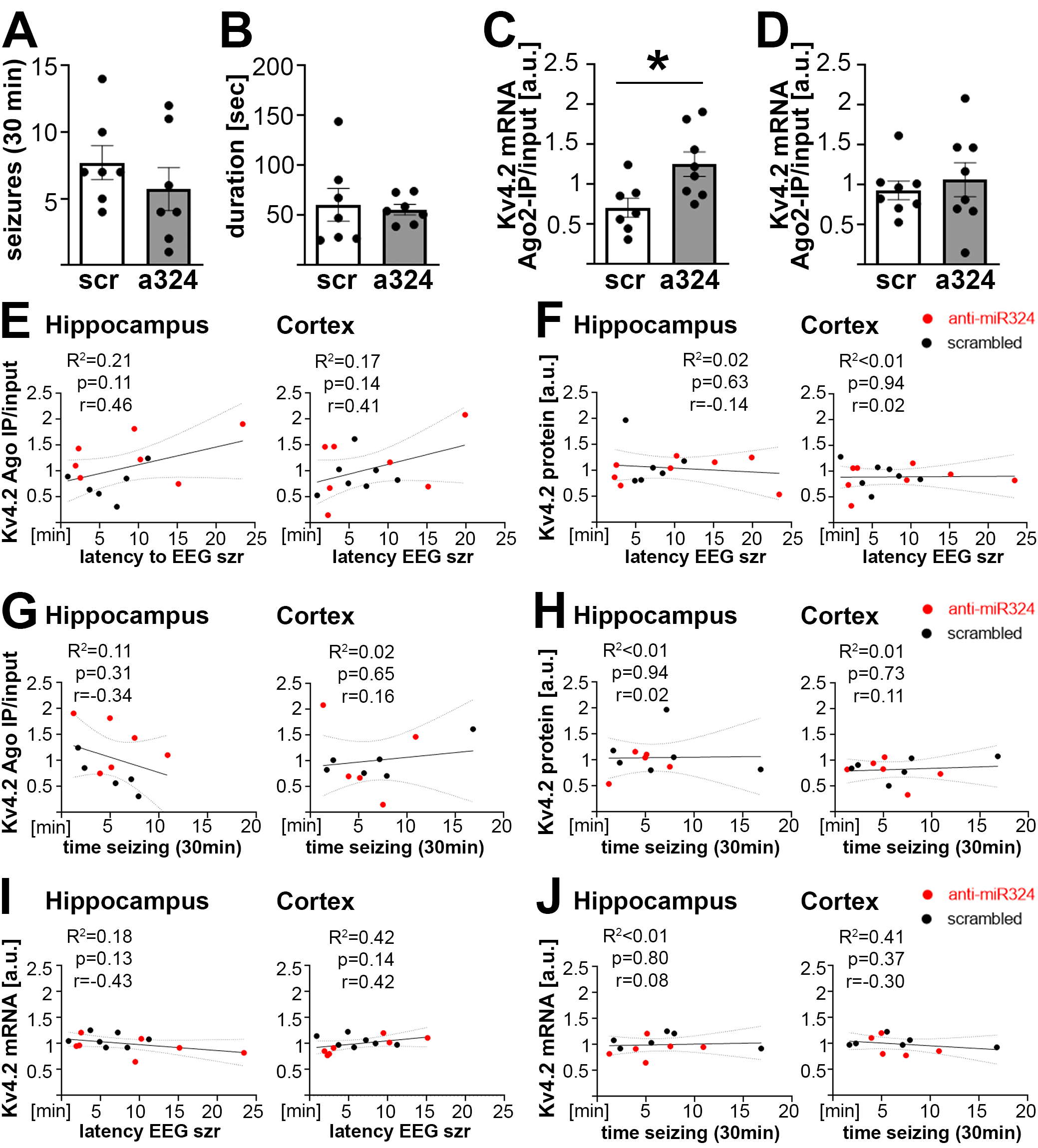

Supplement: Figure 2-1 — MiR-324-5p inhibition does not change numbers or duration of seizures after kainic acid treatment in female mice, and miRNA-induced silencing of Kv4.2 does not correlate with seizure severity. A, B, Number (A) and duration (B) of seizures within the first 30 min after kainic acid injection are not significantly changed after treatment with miR-324-5p antagomirs compared with scrambled controls (unpaired t tests, n = 7; A, p = 0.35; B, p = 0.79). C, D, Association of Kv4.2 mRNA with the RISC is significantly increased after kainic acid-induced seizure in miR-324-5p antagomir-treated mice compared with scrambled antagomir-treated mice in the hippocampus (C), but no the cortex (D; unpaired t tests, n = 7; C, p = 0.965). Bars and error bars represent the mean ± SEM. E–H, No significant correlations of Kv4.2 mRNA RISC association (E, G) and Kv4.2 protein (F, H) in hippocampus or cortex with latency to seizure (E, F) or time seizing (G, H; Pearson’s correlations; E: hippocampus: r = 0.46, R2 = 0.21, p = 0.11; n = 13; cortex: r = 0.41, R2 = 0.17, p = 0.14; n = 14; F: hippocampus: r = –0.14, R2 = 0.02, p = 0.63; n = 14; cortex: r = 0.02, R2 < 0.001, p = 0.94; n = 15; G: hippocampus: r = –0.34, R2 = 0.11, p = 0.31; n = 11; cortex: r = 0.16, R2 = 0.02, p = 0.65; n = 11; H: hippocampus, r = 0.02, R2 < 0.01, p = 0.94; n = 11; cortex: r = 0.11, R2 = 0.01, p = 0.73; n = 12). I, J, Likewise, no significant correlations between hippocampal or cortical Kv4.2 mRNA and latency to seizure (I; Pearson’s correlations; hippocampus: r = –0.43, R2 = 0.18, p = 0.13; n = 14; cortex: r = 0.42, R2 = 0.42, p = 0.14; n = 13) or time seizing (J; Pearson’s correlations; hippocampus: r = 0.08, R2 < 0.01, p = 0.80; n = 12; cortex: r = –0.30, R2 = 0.41, p = 0.37; n = 11). Dashed lines indicate 95% confidence intervals. MiR-324-5p antagomir-treated mice are represented as red dots, and scrambled antagomir-treated mice as black dots. Pearson’s correlation statistics are also shown in the graphs in E–J. [file enu-eN-NWR-0047-22-s03.tif]

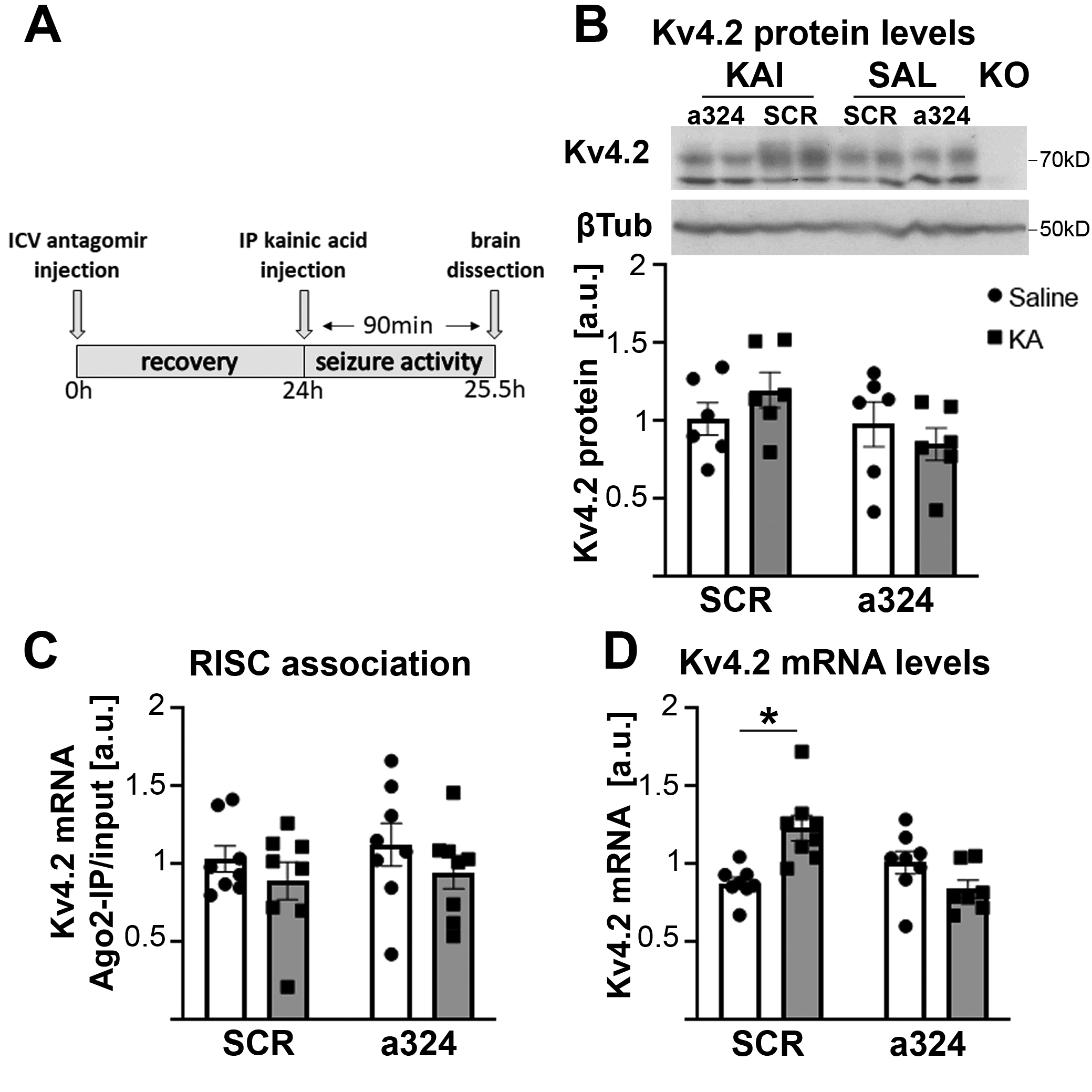

Supplement: Figure 3-1 — Stereotaxically delivered antagomirs combined with kainic acid-induced seizures do not change Kv4.2 protein levels or RISC association of Kv4.2 mRNA in the cortex of female mice but change Kv4.2 mRNA levels. A, Illustration depicting the experimental timeline. MiR-324-5p-specific or scrambled antagomirs were stereotaxically intracerebroventricularly injected. Twenty-four hours later, 15 mg/kg kainic acid was intraperitoneally injected and brains were dissected 90 min later. B, Kv4.2 protein levels were not significantly changed in the cortex of female mice by antagomir or kainic acid treatment (2-way ANOVA; interaction: F(1,20) = 0.04, p = 0.846; effect of antagomir: F(1,20) = 0.34, p = 0.564; effect of kainic acid: F(1,20) = 0.28, p = 0.61; n = 6/group). Kv4.2 protein levels were normalized to β3-tubulin levels on the same blot. C, Likewise, RISC association of Kv4.2 mRNA was not significantly changed in the cortex of female mice by antagomir or kainic acid treatment (2-way ANOVA; interaction: F(1,28) = 0.03, p = 0.873; effect of antagomir: F(1,28) = 0.42, p = 0.521; effect of kainic acid: F(1,28) = 2, p = 0.168; n = 8). D, In contrast, Kv4.2 mRNA levels were significantly increased by seizure in the cortex of scrambled antagomir-injected female mice, but not in those of miR-324-5p-specific antagomir-injected female mice (2-way ANOVA with Dunnett’s multiple-comparison tests; interaction: F(1,27) = 16.4, *p = 0.004; effect pf antagomir: F(1,27) = 4.02, p = 0.0.055; effect of kainic acid: F(1,27) = 2, p = 0.168, *p = 0.0015; n = 8, except for a324/kA, n = 7). Kv4.2 mRNA levels were normalized to Gapdh mRNA. a.u., Arbitrary units. Bars and error bars represent the mean ± SEM. Analyses in hippocampal tissue are shown in Figure 3. Download Figure 3-1, TIF file. [file enu-eN-NWR-0047-22-s04.tif]

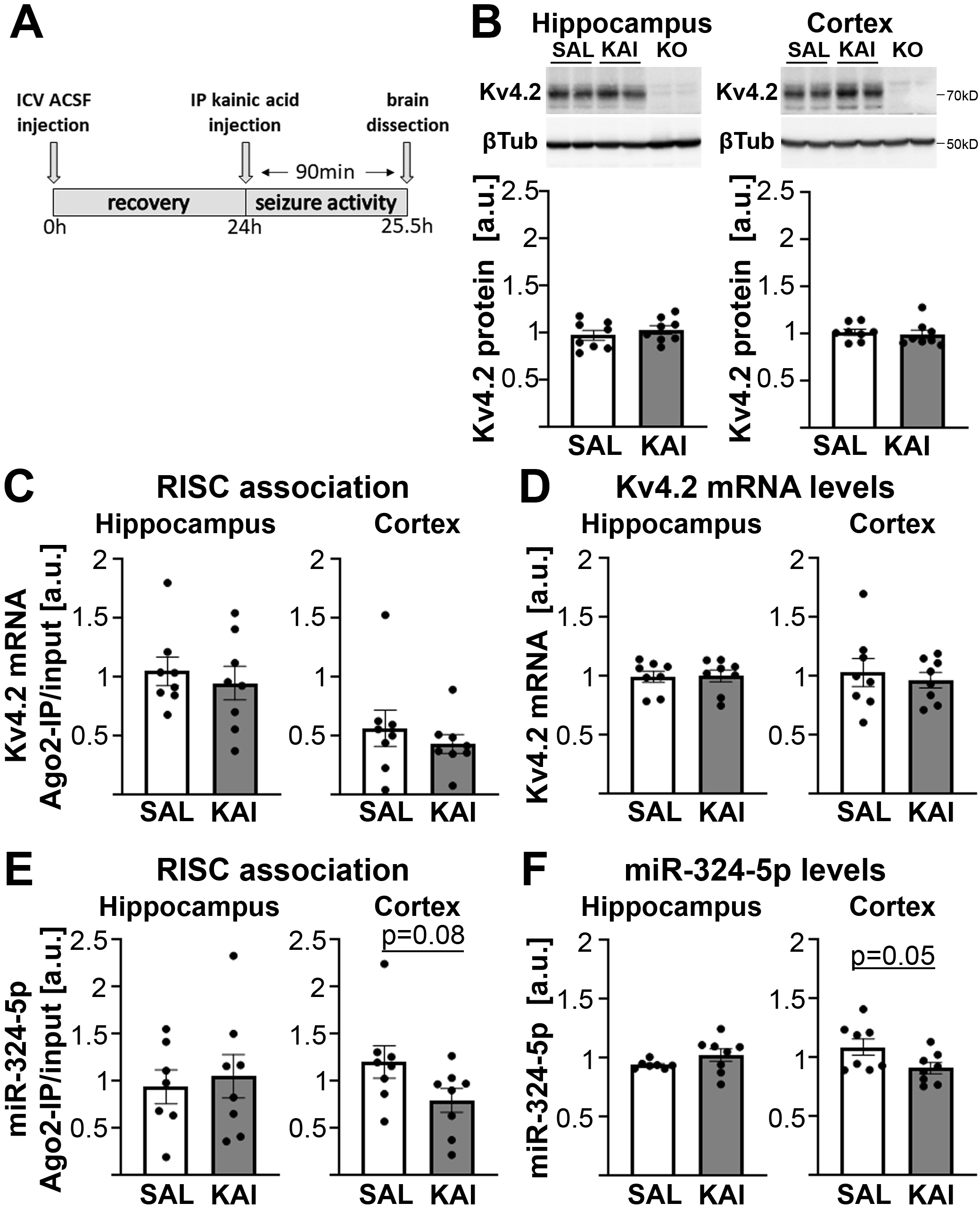

Supplement: Figure 3-2 — Sham surgeries blunt the effect of kainic acid-induced seizures on Kv4.2 protein levels in female mice. A, Illustration depicting the experimental timeline. ACSF (vehicle) was stereotaxically intracerebroventricularly injected. Twenty-four hours later, 15 mg/kg kainic acid was intraperitoneally injected, and brains were dissected 90 min later. B, Kv4.2 protein levels were not significantly changed in the hippocampus or cortex of female mice by kainic acid treatment 24 h after a sham surgery (hippocampus: unpaired t test, p = 0.413; n = 8; cortex: Mann–Whitney test, p = 0.593; n = 8). Kv4.2 protein levels were normalized to β3-tubulin levels on the same blot. Example blots shown on top. C, Likewise, RISC association of Kv4.2 mRNA was not significantly changed in the hippocampus or cortex of sham female mice by kainic acid treatment (unpaired t tests: p(hippocampus) = 0.598, p(cortex) = 0.460; n = 8). D, Kv4.2 mRNA levels were unchanged in hippocampus or cortex of sham intracerebroventricularly injected female mice after kainic acid-induced seizures (unpaired t tests: p(hippocampus) = 0.932, p(cortex) = 0.633; n = 8). Kv4.2 mRNA levels were normalized to Gapdh mRNA. E, RISC association of miR-324-5p was not significantly changed in the hippocampus or cortex of sham female mice by kainic acid treatment (unpaired t tests: p(hippocampus) = 0.714, n(SAL) = 7, n(KAI) = 8; p(cortex) = 0.076; n = 8). F, MiR-324-5p levels were unchanged in hippocampus of sham intracerebroventricularly injected female mice after kainic acid-induced seizures, but significantly reduced in the cortex (unpaired t tests; hippocampus: p = 0.176; n(SAL) = 7, n(KAI) = 8; one statistical outlier removed from the saline group; cortex: unpaired t test: p = 0.050, n = 8). MiR-324-5p levels were normalized to miR-191. a.u., Arbitrary units. Bars and error bars represent the mean ± SEM. Analyses of antagomir-injected mice in hippocampal and cortical tissue are shown in Figure 3 and Extended Data Figure 3-1, [file enu-eN-NWR-0047-22-s05.tif]

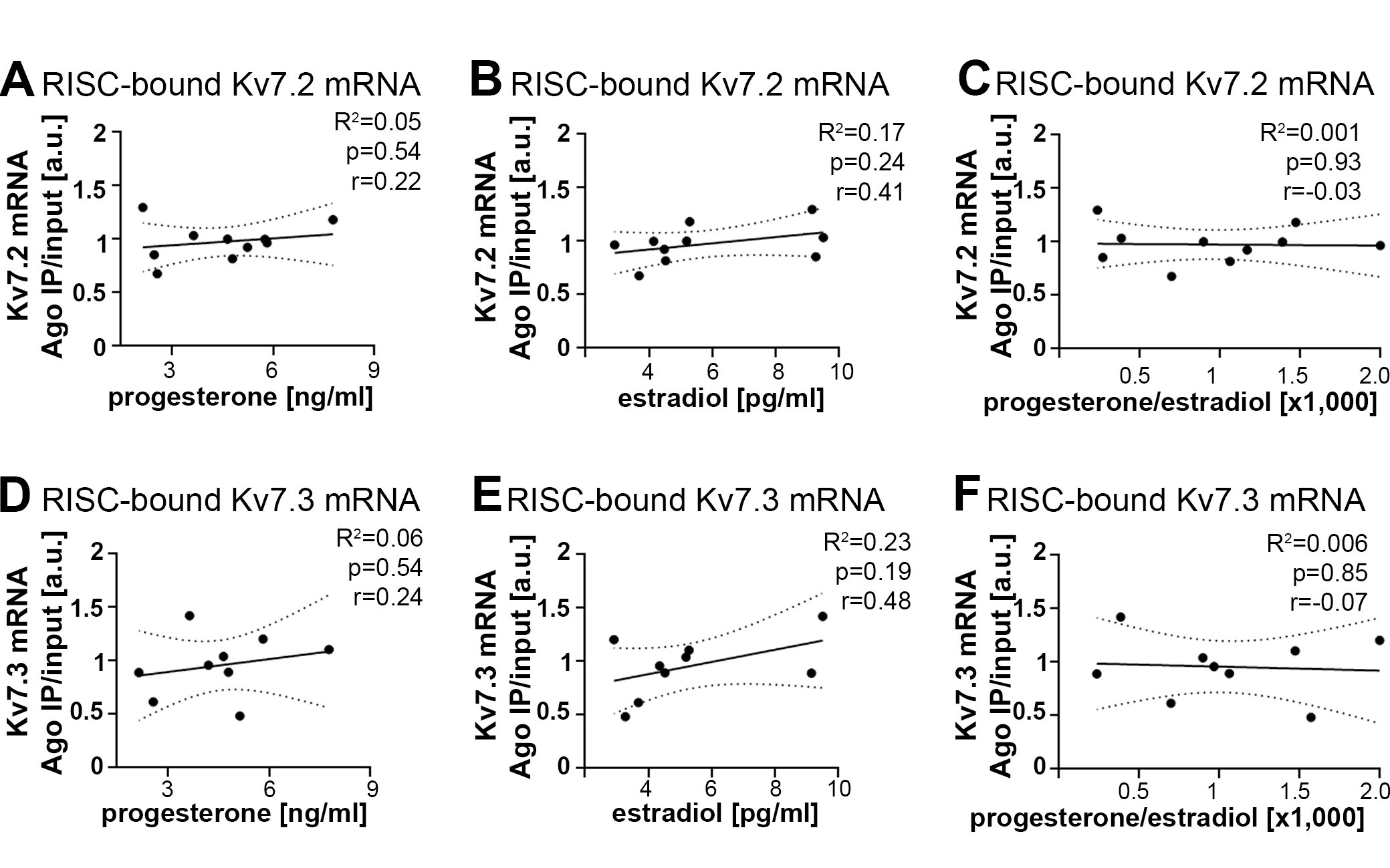

Supplement: Figure 4-1 — RISC association of the voltage-gated potassium channels Kv7.2 and Kv7.3 in the hippocampus are not correlated with plasma levels of progesterone or 17β-estradiol. A–C, Kv7.2 mRNA association with the RISC in hippocampal lysates from female mice is not significantly correlated with plasma levels of progesterone (A; Pearson’s correlation: r = 0.22, R2 = 0.05, p = 0.54; n = 10), 17β-estradiol (B; Pearson’s correlation: r = 0.41, R2 = 0.17, p = 0.24; n = 10), and progesterone/17β-estradiol ratios (C; Pearson’s correlation: r = –0.03, R2 = 0.001, p = 0.93; n = 10). D–F, Likewise, RISC association of Kv7.3 mRNA is not significantly correlated with progesterone (D; Pearson’s correlation: r = 0.24, R2 = 0.06, p = 0.54; n = 9), 17β-estradiol (E; Pearson’s correlation: r = 0.48, R2 = 0.23, p = 0.19; n = 9), or progesterone/17β-estradiol ratios (F; Pearson’s correlation: r = –0.07, R2 = 0.006, p = 0.85; n = 9). Dashed lines indicate 95% confidence intervals. Pearson’s correlation statistics are also shown in the figure. Corresponding analyses of Kv4.2 are shown in Figure 4. Download Figure 4-1, TIF file. [file enu-eN-NWR-0047-22-s06.tif]

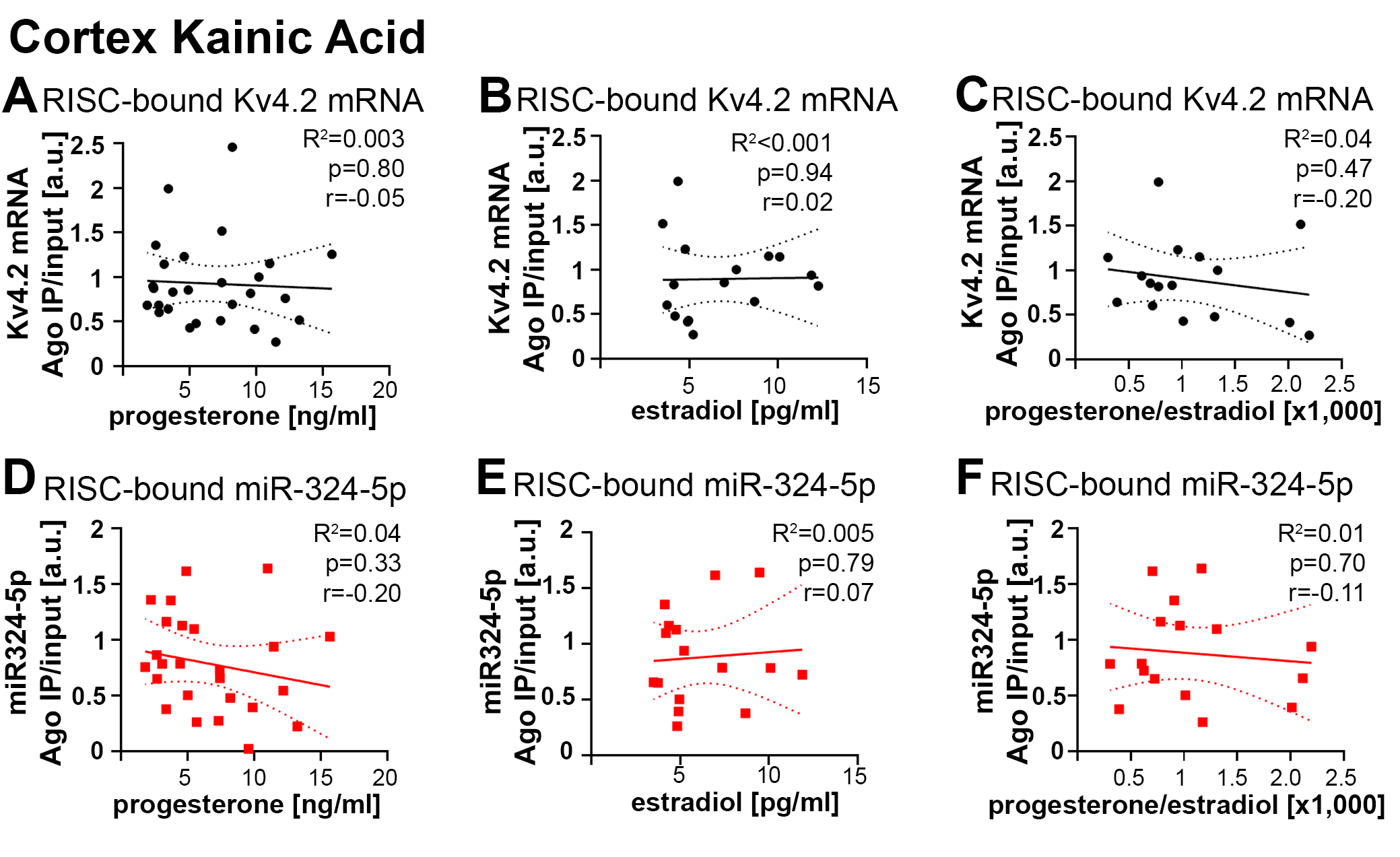

Supplement: Figure 6-1 — In the cortex, treatment with kainic acid abolishes all significant correlations of microRNA-induced silencing of Kv4.2 with plasma levels of progesterone and 17β-estradiol. A–C, Ninety minutes following kainic acid treatment, Kv4.2 mRNA association with the RISC in cortical lysates from female mice is not significantly correlated with plasma levels of progesterone (A; Pearson’s correlation: r = –0.05, R2 = 0.003, p = 0.80; n = 27), 17β-estradiol (B; Pearson’s correlation: r = 0.02, R2 = 0.0004, p = 0.94; n = 16), and progesterone/17β-estradiol ratios (C; Pearson’s correlation: r = –0.20, R2 = 0.04, p = 0.47; n = 16). D–F, Likewise, no significant correlations between RISC association of miR-324-5p with plasma progesterone (D; Pearson’s correlation: r = –0.20, R2 = 0.04, p = 0.33; n = 25), 17β-estradiol was detected (E, Pearson’s correlation: r = 0.07, R2 = 0.005, p = 0.79; n = 16), and progesterone/17β-estradiol ratios (F; Pearson’s correlation: r = –0.11, R2 = 0.01, p = 0.70; n = 16) was observed. Dashed lines indicate 95% confidence intervals. Pearson’s correlation statistics are also shown in the figure. Analyses in hippocampal tissue are shown in Figure 6. Download Figure 6-1, TIF file. [file enu-eN-NWR-0047-22-s07.tif]

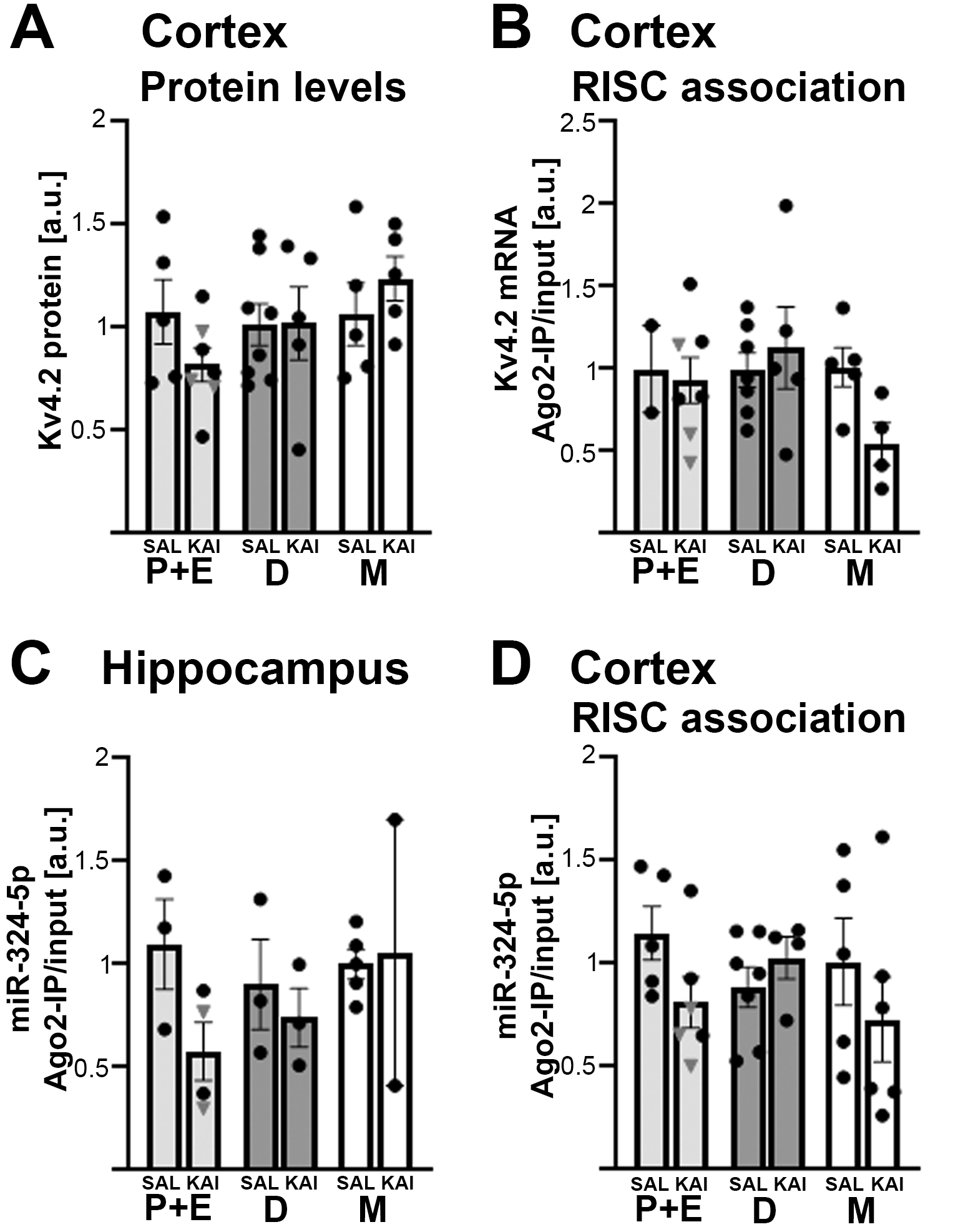

Supplement: Figure 7-1 — No significant differences in miR-324-5p association with the RISC in hippocampus or cortex, and no changes in Kv4.2 protein expression and Kv4.2 mRNA association with the RISC in the cortex across estrous cycle stages and depending on kainic acid treatment. A, Kv4.2 protein levels in the cortex not significantly different across estrous stages in female saline-treated or kainic acid-treated mice (two-way ANOVA; interaction: F(2,29) = 1.4, p = 0.26; effect of estrous stage: F(2,29) = 1.2, p = 0.307; effect of kainic acid: F(1,29) = 0.06, p = 0.807; n(proestrus+estrus saline) = 5, n(proestrus+estrus kainic acid) = 7, n(diestrus saline) = 8, n(diestrus kainic acid) = 5, n(metestrus saline) = 5, and n(metestrus kainic) = 5). Kv4.2 was normalized to β3-tubulin signal on the same blot. B, Kv4.2 mRNA association with the RISC in the cortex is not significantly different across estrous stages in female saline-treated or kainic acid-treated mice (two-way ANOVA; interaction: F(2,24) = 1.8, p = 0.19; effect of estrous stage: F(2,24) = 1.5, p = 0.23; effect of kainic acid: F(1,24) = 0.8, p = 0.37; n(proestrus+estrus saline) = 2, n(proestrus+estrus kainic acid) = 7, n(diestrus saline) = 7, n(diestrus kainic acid) = 5, n(metestrus saline) = 5, and n(metestrus kainic) = 4). C, MiR-324-5p association with the RISC in the hippocampus is not significantly different across estrous stages in female saline-treated or kainic acid-treated mice (two-way ANOVA; interaction: F(2,14) = 1, p = 0.39; effect of estrous stage: F(2,14) = 0.58, p = 0.57; effect of kainic acid: F(1,14) = 1.5, p = 0.24; n(proestrus+estrus saline) = 3, n(proestrus+estrus kainic acid) = 4, n(diestrus saline) = 3, n(diestrus kainic acid) = 3, n(metestrus saline) = 5, and n(metestrus kainic) = 2). D, MiR-324-5p association with the RISC in the cortex is not significantly different across estrous stages in female saline-treated or kainic acid-treated mice (two-way ANOVA; interaction: F(2,27) = 1.4, p = 0.272; effect of e [file enu-eN-NWR-0047-22-s08.tif]
